# CD38–RyR2 axis–mediated signaling impedes CD8^+^ T cell response to anti-PD1 therapy in cancer

**DOI:** 10.1073/pnas.2315989121

**Published:** 2024-03-07

**Authors:** Anwesha Kar, Puspendu Ghosh, Anupam Gautam, Snehanshu Chowdhury, Debashree Basak, Ishita Sarkar, Arpita Bhoumik, Shubhrajit Barman, Paramita Chakraborty, Asima Mukhopadhyay, Shikhar Mehrotra, Senthil Kumar Ganesan, Sandip Paul, Shilpak Chatterjee

**Affiliations:** a Division of Cancer Biology and Inflammatory Disorder, Translational Research Unit of Excellence, Council of Scientific and Industrial Research-Indian Institute of Chemical Biology, Kolkata 700032, West Bengal, India; b Academy of Scientific and Innovative Research (AcSIR), Ghaziabad 201002, Uttar Pradesh, India; c Algorithms in Bioinformatics, Institute for Bioinformatics and Medical Informatics, University of Tübingen, Sand 14 72076, Tübingen, Baden-Württemberg, Germany; d International Max Planck Research School “From Molecules to Organisms”, Max Planck Institute for Biology Tübingen 72076, Tübingen, Baden-Württemberg, Germany; e Division of Structural Biology & Bioinformatics, Council of Scientific and Industrial Research-Indian Institute of Chemical Biology, Kolkata 700032, West Bengal, India; f Department of Surgery, Medical University of South Carolina, Charleston, South Carolina SC-29425; g Kolkata Gynaecology Oncology Trials and Translational Research Group, Kolkata 700156, West Bengal, India; h System Biology Informatics Lab, Center for Health Science and Technology, JIS Institute of Advanced Studies and Research, JIS University, Kolkata 700091, West Bengal, India

**Keywords:** T cell exhaustion, anti-PD1 resistance, CD38

## Abstract

PD1 blockade therapy, harnessing the cytotoxic potential of CD8^+^ T cells, has yielded clinical success in treating malignancies. However, its efficacy is often limited due to the progressive differentiation of intratumoral CD8^+^ T cells into a hypofunctional state known as terminal exhaustion. Despite identifying CD8^+^ T cell subsets associated with immunotherapy resistance, the molecular pathway triggering the resistance remains elusive. Given the clear association of CD38 with CD8^+^ T cell subsets resistant to anti-PD1 therapy, we investigated its role in inducing resistance. Phenotypic and functional characterization, along with single-cell RNA sequencing analysis of both in vitro chronically stimulated and intratumoral CD8^+^ T cells, revealed that CD38-expressing CD8^+^ T cells are terminally exhausted. Exploring the molecular mechanism, we found that CD38 expression was crucial in promoting terminal differentiation of CD8^+^ T cells by suppressing TCF1 expression, thereby rendering them unresponsive to anti-PD1 therapy. Genetic ablation of CD38 in tumor-reactive CD8^+^ T cells restored TCF1 levels and improved the responsiveness to anti-PD1 therapy in mice. Mechanistically, CD38 expression on exhausted CD8^+^ T cells elevated intracellular Ca^2+^ levels through RyR2 calcium channel activation. This, in turn, promoted chronic AKT activation, leading to TCF1 loss. Knockdown of RyR2 or inhibition of AKT in CD8^+^ T cells maintained TCF1 levels, induced a sustained anti-tumor response, and enhanced responsiveness to anti-PD1 therapy. Thus, targeting CD38 represents a potential strategy to improve the efficacy of anti-PD1 treatment in cancer.

Tumor epitope reactive CD8^+^ T cells, despite their high abundance at the tumor site, often fail to control tumor growth, indicating their dysfunctionality (1). This dysfunction, triggered by persistent antigen exposure and subsequently aggravated by hypoxia and metabolic stress, gradually drives intratumoral CD8^+^ T cells into a functionally suppressive state termed T cell exhaustion (2–5). Exhausted CD8^+^ T cells (Tex), characterized by the loss of effector cytokine production (IFN-γ, TNF-α, IL-2), reduced proliferative capacity, and elevated cell surface expression of various co-inhibitory receptors (such as PD1, CTLA4, Tim3, Lag3, TIGIT), are unable to mount an effective anti-tumor response (3). In recent years, immune checkpoint blockade (ICB) therapy targeting PD1, aimed at reinvigorating the effector function of the Tex cells in tumors, has achieved unprecedented clinical success in treating various cancers (6). Nevertheless, the therapeutic efficacy remains confined to a small subset of patients with malignancies, and moreover, in the majority of patients, the response is transient (7, 8).

Tex cells are heterogeneous (9). The heterogeneity in Tex cells arises due to their gradual transition from a less differentiated state to a terminal differentiation state, leading to variations in functionality across their developmental trajectory (10). Progenitor-exhausted T cells (Tpex), carrying proliferative and self-renewal capacity maintained by TCF1 expression, respond to anti-PD1 therapy and mediate tumor control (11–13). In contrast, the progression of Tpex cells to terminally exhausted T cells (Tex^term^), characterized by the impaired proliferative potential and effector function, are non-responsive to ICB therapy (11, 14). The most prominent change accompanying the progression of Tpex to Tex^term^ is the concomitant expression of multiple co-inhibitory and co-stimulatory receptors believed to have distinct roles in impairing the overall functionality of Tex^term^ cells (9, 15, 16). However, the precise contribution of any such receptor to ICB resistance development is yet to be deciphered.

Recent research efforts aimed at comprehensively characterizing Tex subsets predictive of immunotherapy resistance have identified the PD1^+^CD38^hi^CD8^+^ T cell subset, highly abundant in both peripheral blood and tumor tissue of patients resistant to ICB therapy (17).

Subsequent validation in pre-clinical models further confirmed that intratumoral PD1^+^CD38^hi^CD8^+^ T cells are dysfunctional and directly contribute to anti-PD1 resistance (17). Interestingly, an earlier study reported that CD38, in conjunction with CD101, marks the intratumoral PD1^hi^ CD8^+^ T cells as a non-reprogrammable subset refractory to functional rejuvenation (18). Recently, a CD4^+^ T cell subset co-expressing CD38 and CD39 was also observed in metastatic melanoma patients resistant to anti-PD1 therapy (19). These findings underscore the critical association of CD38 expression with intratumoral T cell subsets not amenable to functional rejuvenation by ICB therapy. CD38, a transmembrane glycoprotein, possesses an enzymatic role in metabolizing NAD^+^ and catalyzing the generation of cyclic adenosine 5′-diphosphate ribose (cADPR) (20). Recently, the role of CD38 has been implicated in regulating various aspects of T cell metabolism and effector functions (21). However, whether CD38 has any direct influence in regulating the responsiveness of Tex cells to anti-PD1 therapy, and if so, the underlying mechanism has remained unanswered.

In this study, we conducted phenotypic, functional, and single-cell RNA sequencing (scRNA-seq)-based transcriptome profiling of CD8^+^ T cells obtained from either the tumor site or those chronically stimulated in vitro. The results established CD38 as an important immune checkpoint associated with the Tex subset, exhibiting characteristic features of Tex^term^ cells. Functionally, the expression of CD38 on Tex cells orchestrates a signaling cascade by elevating intracellular Ca^2+^ levels, leading to the suppression of TCF1 expression and, consequently, developing resistance to anti-PD1 therapy. Thus, targeting CD38 along with PD1 could be a potential strategy to improve the efficacy of cancer immunotherapy.

## Results

### CD38 Expression is Associated with Intratumoral CD8^+^ T cells Exhibiting Terminal Differentiation.

**Fig. 1:**
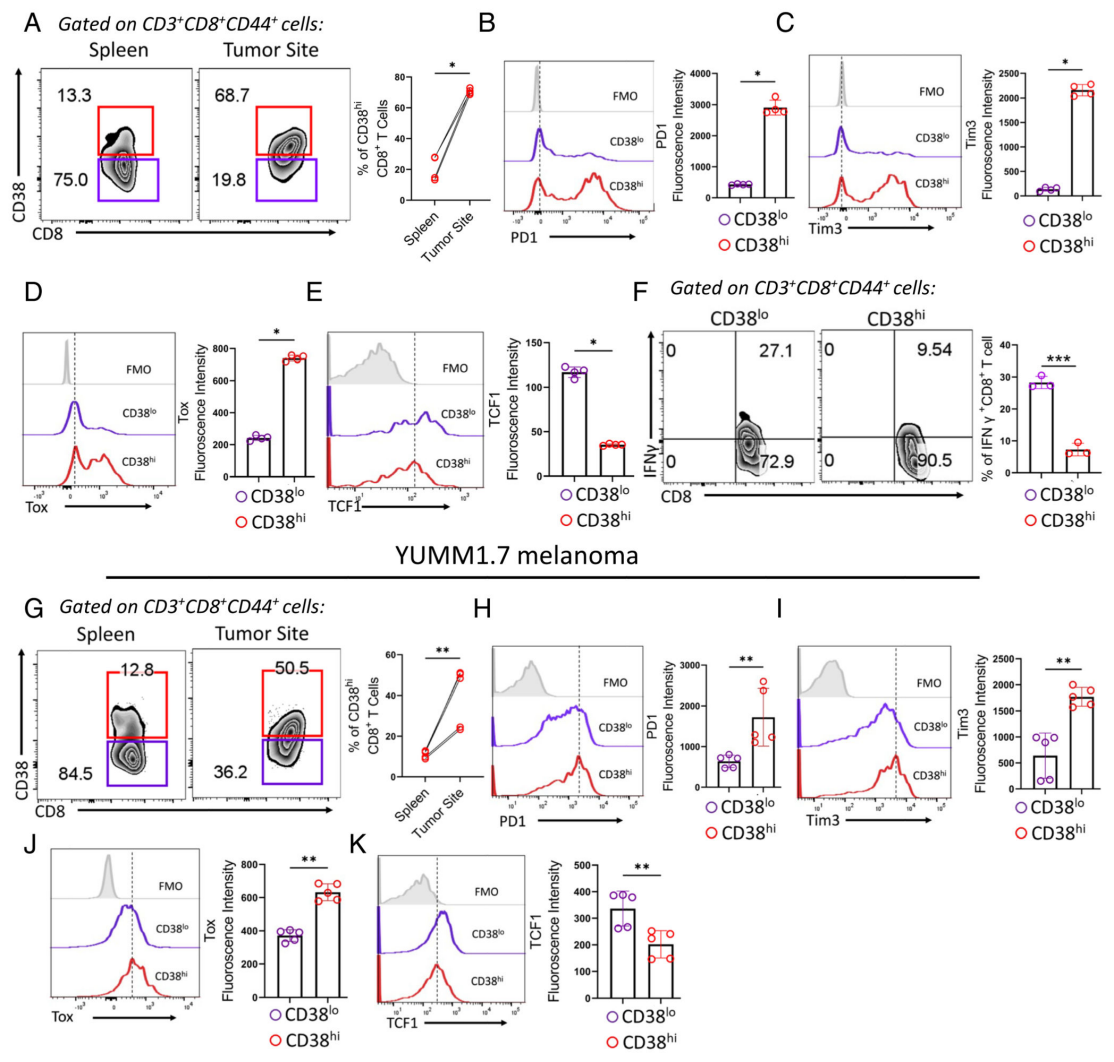
Phenotypic and functional characterization of intratumoral CD38-expressing CD8^+^ T cells. (*A*) Frequency of CD38^hi^ CD8^+^ T cells in B6 mice bearing 15 d subcutaneously established B16-F10 melanoma. The scatter plot represents the cumulative data of four independent experiments. (*B*–*E*) Expression of (*B*) PD1, (*C*) Tim3, (*D*) Tox, and (*E*) TCF1 in intratumoral CD8^+^ T cell stratified based on CD38 expression. Adjacent bar plots represent cumulative data of median fluorescence intensity from four independent experiments. (*F*) Intracellular IFNγ production by intratumoral CD38^hi^CD8^+^ and CD38^lo^CD8^+^ T cells. The bar plot is representative of three independent experiments. (*G*) Abundance of CD38^hi^ CD8^+^ T cells in B6 mice bearing 15 d subcutaneously established murine YUMM1.7 melanoma model. The adjacent scatter plot represents the cumulative data of five independent experiments. (*H*–*K*) Intratumoral CD38^hi^ and CD38^lo^ CD8^+^ T cells from YUMM1.7 bearing mice were assessed for (*H*) PD1, (*I*) Tim3, (*J*) Tox, and (*K*) TCF1. Adjacent bar plots represent cumulative data of median (*H*–*J*) or mean (*K*) fluorescence intensity from five independent experiments. *P < 0.05; **P < 0.01; ***P < 0.005; ****P < 0.0001; ns, nonsignificant.

To begin to delineate the phenotypic and functional traits associated with CD38-expressing CD8^+^ T cells and their involvement in anti-PD1 resistance, we employed the highly aggressive B16-F10 melanoma tumor model, which has shown resistance to immunotherapy. First, we evaluated the abundance of tumor antigen-experienced (CD44^hi^) CD38-expressing CD8^+^ T cells in the tumor-bearing host when the tumor size reached ~100 to 120 mm^2^ (between day 14 and day 15). We observed that the frequency of CD38-expressing CD8^+^ T cells was ~threefold to fourfold more abundant at the tumor site than the spleen (Fig. 1*A* and SI Appendix, Fig. S1*A*). We next wanted to determine the exhaustion state of these cells by further analyzing the expression of various pre-defined exhaustion-associated markers. Intratumoral CD38-expressing CD8^+^ T cells were characterized as Tex^term^ cells, considering the remarkably high expression of PD1 (Fig. 1*B*) and Tim3 (Fig. 1*C*), elevated Tox levels (Fig. 1*D*), reduced expression of TCF1 (Fig. 1*E*), and failure to secrete IFNγ upon restimulation (Fig. 1*F*).

We further validated our findings in another immunotherapy-resistant tumor model, YUMM1.7, a mouse melanoma line containing human-relevant mutations (Braf^V600E^; Cdkn2a^−/−^ Pten^−/−^; Tyrosinase:CreERT2). Akin to the B16-F10 tumor model, CD38-expressing CD8^+^ T cells were highly abundant at the tumor site (Fig. 1*G*) and were characterized by the co-expression of PD1 (Fig. 1*H*) and Tim3 (Fig. 1*I*), elevated expression of Tox (Fig. 1*J*), and reduced TCF1 levels (Fig. 1*K*). These findings collectively emphasize the exclusive association of CD38 with intratumoral CD8^+^ T cells displaying the hallmark characteristics of terminally differentiated Tex cells, which are non-reprogrammable and hyporesponsive to immunotherapy.

### Chronic TCR Stimulation of Human CD8^+^ T cells Drives the Expression of CD38 on Tex Cells

**Fig. 2:**
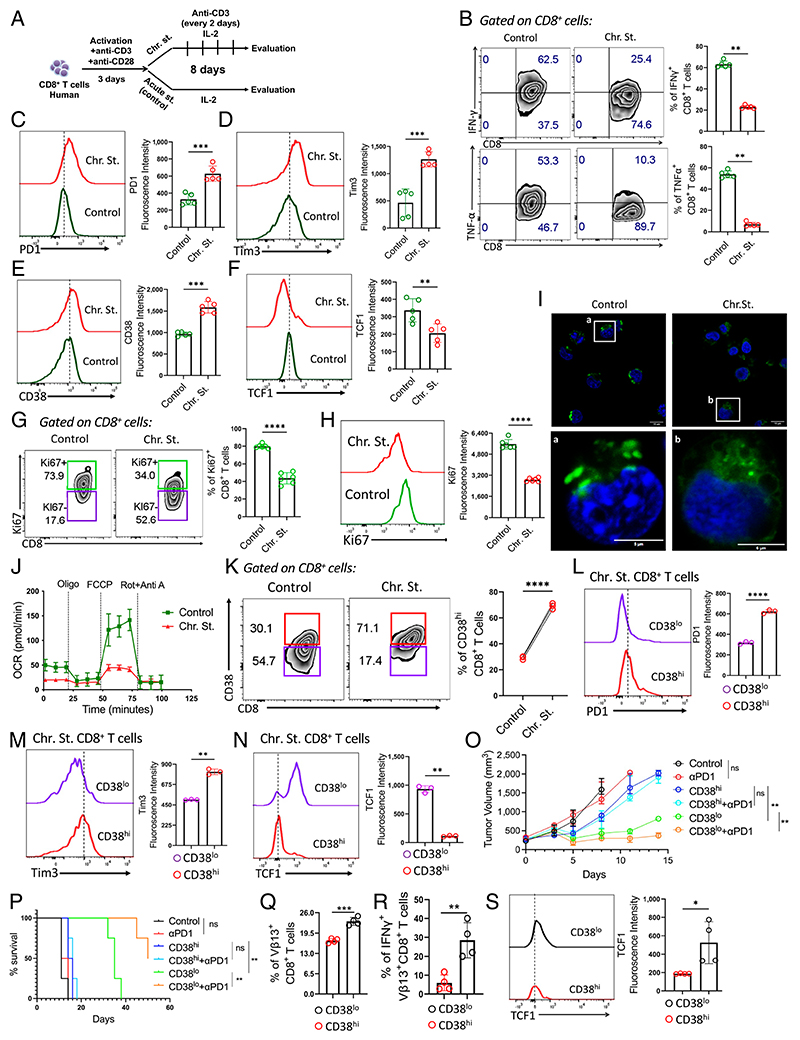
Chronic TCR stimulation drives the expression of CD38 on T cells. (*A*) Schematic diagram representing the protocol used for generating control or chronic CD8^+^ T cells from healthy human donors. (*B*–*H*) Control or chronic CD8^+^ T cells were assessed for (*B*) production of intracellular cytokines, expression of (*C*) PD1, (*D*) Tim3, (*E*) CD38, (*F*) TCF1, (*G*) frequency of Ki67^+^ CD8 T cells, and (*H*) Ki67 expression. (*I*) Confocal microscopic image representing distinct mitochondrial morphology stained with MitoTracker Green (in green). Nuclei were stained with DAPI (in blue). The *Lower* panels show the magnified images of the cells in the white box drawn in the *Upper* panels. (*J*) Oxygen consumption rate (OCR) under basal condition and in response to indicated mitochondrial inhibitors. (*K*) Control and chronically stimulated CD8^+^ T cells were evaluated for the frequency of CD38^hi^ CD8^+^ T cells. (*L*–*N*) Expression of (*L*) PD1, (*M*) Tim3, and (*N*) TCF1 in CD38^hi^ and CD38^lo^ CD8^+^ T cells obtained from chronically stimulated CD8^+^ T cells. (*O*) Assessment of tumor growth after adoptive transfer of CD38^hi^ and CD38^lo^ Pmel T cells, either with control IgG or in combination with anti-PD1 antibody in mice bearing B16-F10 melanoma tumor. (*P*) KM curves for time-to-killing for experimental conditions are shown. (*Q*–*S*) Adoptively transferred Vβ13^+^ Pmel T cells retrieved from the tumor site were assessed for (*Q*) persistence, (*R*) intracellular IFNγ production, and (*S*) expression of TCF1. Bar plots adjacent to figures represent cumulative data of (*B*) frequency of cytokine-positive cells from five, (*C*–*F*) mean fluorescence intensity from five, (*G*) frequency of cells from six, (*H*) mean fluorescence intensity from six, (*J*) three, (*K*) frequency of cells from three, (*L* and *M*) mean fluorescence intensity from three, (*N*) median fluorescence intensity from three, and (*S*) mean fluorescence intensity from four independent experiments. *P < 0.05; **P < 0.01; ***P < 0.005; ****P < 0.0001; ns, nonsignificant.

To elucidate whether elevated expression of CD38 on Tex cells results from chronic TCR stimulation, T cells isolated from healthy human PBMC were activated for 3 d (with anti-CD3+antiCD28) followed by their in vitro expansion in the absence (control) or presence of chronic antigenic stimulation (chronic) for another 8 d (Fig. 2*A*). To determine whether chronic TCR stimulation of human CD8^+^ T cells using these protocols induced T cell exhaustion, we checked their effector cytokines production and observed a dramatic reduction in both IFNγ and TNFα production (Fig. 2*B*). Moreover, chronically stimulated human CD8^+^ T cells also displayed increased expression of various immune checkpoint molecules, including CD38 (Fig. 2 *C*–*E*), compared to the control. In addition, we observed reduced TCF1 expression (Fig. 2*F*), increased susceptibility to apoptosis (SI Appendix, Fig. S1*B*), and impaired proliferation capacity (Fig. 2 *G* and *H*), characteristic hallmarks of Tex cells in the chronically stimulated group. Consistent with the recent study, we also noted a comparable Tox expression between control and chronic Tex cells (SI Appendix, Fig. S1*C*), supporting the notion that in vitro differentiated chronic Tex cells are less dependent on Tox; instead, additional stimuli may be needed to induce and sustain Tox expression (22).

Since Tex cells are often defined by impaired mitochondrial metabolism (4, 5), we assessed mitochondrial integrity and oxidative phosphorylation (OXPHOS). Mitochondria in chronic cells appeared “defused and round shaped” compared to “dense and elongated” in control (Fig. 2*I*). Consistently, oxygen consumption rate, a measure of mitochondrial OXPHOS, was markedly downregulated in chronic cells compared to control (Fig. 2*J*). Together, these data indicate that human CD8^+^ T cells chronically stimulated for 11 d acquire key characteristics of Tex cells.

We, next, stratified in vitro differentiated chronic Tex cells based on their CD38 expression (Fig. 2*K*) and evaluated phenotypic features of CD38^hi^ and CD38^lo^ cells. Similar to in vivo findings, chronic Tex, expressing CD38, had high co-expression of PD1 (Fig. 2*L*) and Tim3 (Fig. 2*M*). Consistently, CD38 exhibited an inverse association with TCF1 expression in chronic Tex cells (Fig. 2*N*).

Next, we evaluated whether Tex cells sorted based on CD38 expression (CD38^hi^ and CD38^lo^ Tex) exerted differential responsiveness to anti-PD1 therapy. Pmel T cells specific for gp100 were chronically stimulated in vitro for 8 d, and then the Vβ13^+^CD8^+^ T cells sorted into CD38^hi^ and CD38^lo^ fraction were adoptively transferred into mice bearing B16-F10 melanoma tumor. Mice who received adoptive T cell therapy (ACT) were treated with either anti-PD1 antibody or control IgG, as outlined in SI Appendix, Fig. S1*D*. Adoptive transfer of CD38^hi^ Pmel T cells failed to control tumor growth, and their therapeutic efficacy could not be improved upon anti-PD1 therapy. Conversely, CD38^lo^ Pmel T cells mounted a robust anti-tumor response, which was further enhanced when ACT was combined with PD1 blockade therapy (Fig. 2 *O* and *P*). Analysis of tumor-infiltrating Vβ13^+^CD8^+^ T cells revealed that the superior tumor control and responsiveness to anti-PD1 therapy by CD38^lo^ T cell fraction could be in part due to their increased persistence (Fig. 2*Q*), better functionality (Fig. 2*R*), and maintenance of high TCF1 expression (Fig. 2*S*), compared to CD38^hi^ T cell subset.

Thus, the characterization of CD8^+^ T cells either isolated from the tumor site or chronically stimulated in vitro convincingly shows that expression of CD38 on Tex cells is primarily associated with the terminally differentiated subset that fails to respond to anti-PD1 therapy.

### CD38-Expressing CD8^+^ T cells Exhibit the Transcriptomic Signature of Tex^term^ Cells.

To define the molecular traits associated with CD38-expressing CD8^+^ T cells, chronically stimulated human CD8^+^ T cells from two different healthy donors, along with T cells expanded without chronic stimulation (Control), were subjected to cellular indexing of Transcriptomes and epitopes by sequencing analysis, where barcoded antibodies against the surface protein PD1 and CD38 were used. We analyzed the scRNA-seq profile of a total of 12,071 T cells (SI Appendix, Fig. S2*A*), of which 6,710 cells were CD8^+^ T cells. Unsupervised clustering analysis of CD8^+^ T cells identified five major clusters (SI Appendix, Fig. S2 *B* and *C*), where clusters 0 and 1 were primarily contributed by CD8^+^ T cells from control cells, and clusters 2, 3, and 4 were mainly due to CD8^+^ T cells from chronic cells (SI Appendix, Fig. S2*D*). Principal component analysis highlighted distinct transcriptional features in chronic cells from control cells (SI Appendix, Fig. S2*E*).

**Fig. 3:**
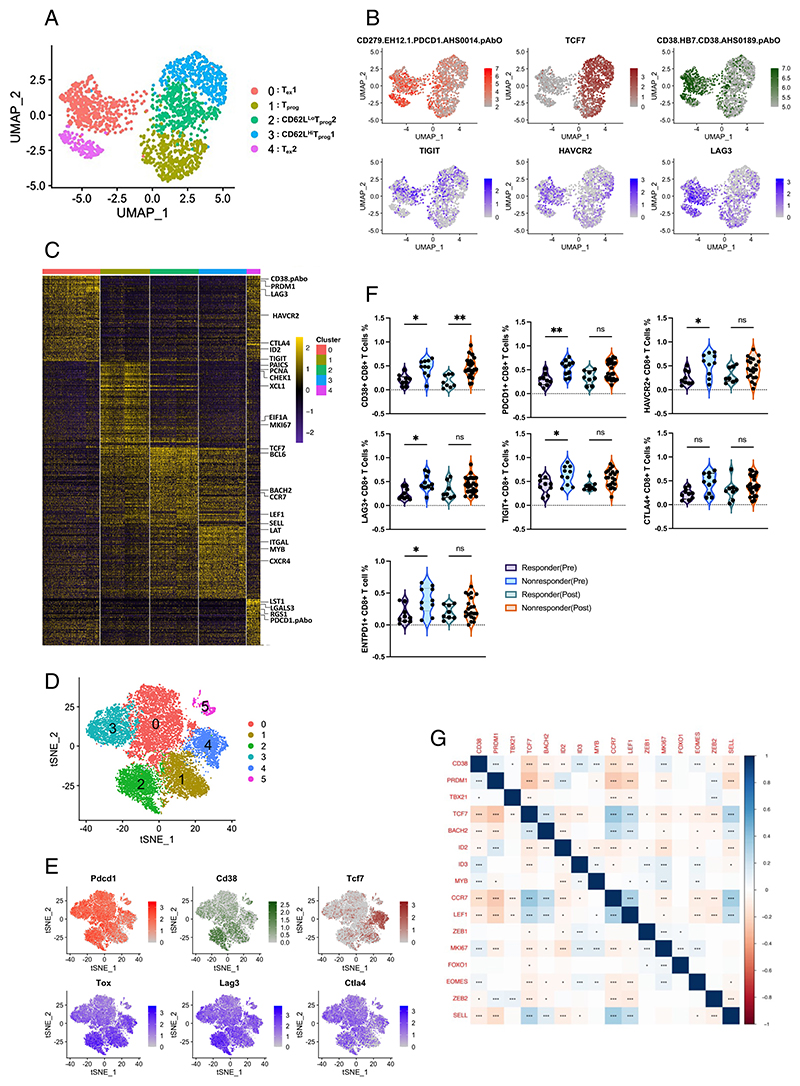
CD38^hi^CD8^+^ T cells exhibit a distinct transcriptomic signature. (*A*) UMAP visualization of the scRNA-seq clusters of chronically stimulated CD8^+^ T cells from 2 donors. (*B*) Single-cell transcription levels of representative genes illustrated in the UMAP plot. Transcription levels and protein expression are color-coded: red (PD1), brown (*TCF7*), and green (CD38); for the *Bottom* panel: blue, expressed; gray, not expressed. (*C*) Heat map showing the top hundred differentially expressed genes (ranked by log2 fold change) of all identified clusters. (*D*) tSNE visualization of the scRNA-seq clusters of murine CD8^+^ T cells from 2 samples: GSE122712 (gp33 tetramer^+^ CD8^+^ T cells isolated on day 28 post-infection from mice chronically infected with Lymphocytic choriomeningitis (LCMV) clone 13 and GSE122675 (tumor infiltrating CD8^+^ T cells from B16-OVA mouse melanoma tumor). (*E*) Single-cell transcription levels of representative genes illustrated in the tSNE plot. Transcription levels are color-coded: red (*Pdcd1*), brown (*Tcf7*), and green (*CD38*); for *Bottom* panel: blue, expressed and gray, not expressed. (*F*) Frequency of respective marker expressing CD8^+^ T cells (1 log normalized count as the threshold) in pre and post-treatment tumor lesions from responder and non-responder groups, as determined by scRNA-seq analysis (One-way ANOVA, *P < 0.05; **P < 0.01; ***P < 0.005; ****P < 0.0001; ns, nonsignificant). (*G*) Spearman correlation plot for genes of interest. Correlation with a *P*-value < 0.05 was considered significant. (*P < 0.05; **P < 0.01; ***P < 0.005; ****P < 0.0001; ns, nonsignificant).

Subsequently, to investigate the transcriptional diversity within the chronically stimulated cells, we re-clustered the CD8^+^ T cells from this group. Uniform manifold approximation and projection (UMAP) analysis based on transcriptomes partitioned chronic CD8^+^ T cells into five clusters (Fig. 3*A*), with varying levels of CD38 and PD1 expression (Fig. 3*B*). We identified two clusters (0 and 4), labeled Tex 1 and Tex 2 cells, characterized by high CD38 expression along with concurrent PD1 expression. Cells in these clusters lack the expression of *TCF7* but are enriched in the expression of inhibitory receptors (*TIGIT, CTLA4, HAVCR2*, and *LAG3*) and exhaustion-associated transcription factors (*ID2* and *PRDM1*) (Fig. 3 *B* and *C*) (10). Moreover, cluster 4 differed from cluster 0 by expressing additional exhaustion-related genes such as RGS1 and LGALS3 (23, 24). In contrast to clusters 0 and 4, the expression level of CD38 was remarkably low in the other three clusters (1, 2, and 3), which predominantly expressed genes associated with either proliferating/effector or progenitor cells (Fig. 3 *B* and *C*). Cluster 1 was characterized as a proliferating subset according to the upregulated levels of several cell-cycle-related genes, including *PCNA, CHEK1, XCL1, MKI67, PAICS*, and *EIF1A*. Consistent with previous study, we identified two precursor T cell (Tex_pre_) clusters (clusters 2 and 3), both are marked by the high expression of *TCF7* and other precursor cell-associated genes, including *CCR7, LEF1, BACH2*, and *BCL6*, but are distinguished by differential expression of *SELL* (encodes CD62L) and hence referred to as CD62L^hi^ Tex_pre_ 1 (cluster 3) and CD62L^lo^ Tex_pre_ 2 (cluster 2) (25). As reported earlier, the CD62L^hi^ Tex_pre_ 1 cluster was enriched for the expression of *MYB* along with other memory-associated transcripts, including *CXCR4, LAT*, and *ITGAL* (25). Furthermore, Gene Ontology (GO) pathway analysis revealed both shared and distinct pathways within each cluster, including cytokine signaling in the immune system, cell activation, metabolism of RNA, cell cycle, and regulation of cell activation (SI Appendix, Fig. S2*F*).

We next sought to determine whether CD8^+^ T cells isolated from mice bearing chronic viral infection and tumor encountered persistent antigen exposure contained analogous subpopulations. We analyzed scRNA-seq datasets of gp33 tetramer^+^ CD8^+^ T cells isolated on day 28 post-infection from mice chronically infected with Lymphocytic choriomeningitis (LCMV) clone 13 (Cl13) and tumor-infiltrating CD8^+^ T cells from B16-OVA mouse melanoma tumor (26). Unsupervised clustering analysis identified six clusters with differential expression levels of *Cd38* (Fig. 3 *D* and *E*). Consistent with our observations in chronically expanded human CD8^+^ T cells, cluster 2, which displayed the transcriptomic signature of Tex^term^ cells according to the upregulated levels of various exhaustion-associated molecules (*Pdcd1, Hacvcr2, Ctla4, Lag3, Cd244, and Entpd1*) and transcription factors (*Tox and Prdm1*), while lacking the expression of *Tcf7*, were highly enriched for the expression of *Cd38* (Fig. 3*E* and SI Appendix, Fig. S2*G*). In contrast, cells in cluster 4 that expressed genes associated with stem-like or progenitor cells, including *Tcf7, Lef1, Id3, Ccr7, Sell, Slamf6*, and *Satb1*, had low expression of *Cd38* (Fig. 3*E* and SI Appendix, Fig. S2*G*). Therefore, transcriptomic analyses support that CD38-expressing Tex cells are in a terminally exhausted state.

Based on the aforementioned findings, we proceeded to delve into the relationship between CD38-expressing CD8^+^ T cells and patients’ responsiveness to ICB therapy. We examined the publicly accessible scRNA-seq data of 48 tumor biopsy samples obtained either pre-or post-treatment from patients (n = 32) with metastatic melanoma treated with anti-PD1 (n = 24) or anti-PD1+anti-CTLA4 (n = 8), as published (9). Our analysis focused on CD8^+^ T cells obtained from both pre-therapy (n = 19) and post-therapy (n = 29) tumor lesions from responder (n = 17) and non-responder (n = 31) patients (SI Appendix, Fig. S3 *A* and *B*). Intriguingly, our findings revealed that, among the different inhibitory receptor-expressing CD8^+^ T cell subsets, CD38^+^CD8^+^ T cells exhibited significantly higher abundance in both pre-therapy (n = 10) and post-therapy (n = 21) non-responding lesions compared to the responding lesions (pre-therapy: n = 9 and post-therapy: n = 8) (Fig. 3*F* and SI Appendix, Fig. S3*C*). Next, to gain insight into the role of CD38, if any, in driving ICB resistance, we examined whether CD38 expression correlated with any pre-defined markers, explicating T cells’ functional and differentiation state (27). Our analysis revealed an inverse correlation between CD38 expression and several memory-associated genes, including *LEF1, CCR7, and SELL* (Fig. 3*G*). Interestingly, the most pronounced negative correlation was observed with *TCF7* expression (Fig. 3*G*), a crucial transcription factor influencing T cell responsiveness to ICB therapy (13). These collective findings shed light on intriguing aspects of CD38-expressing intratumoral CD8^+^ T cells, providing insights into not only their differentiation state but also suggesting a potential role for CD38 in inducing ICB resistance in CD8^+^ T cells.

### Expression of CD38 on Tex Cells Confers Resistance to Anti-PD1 Therapy

**Fig. 4:**
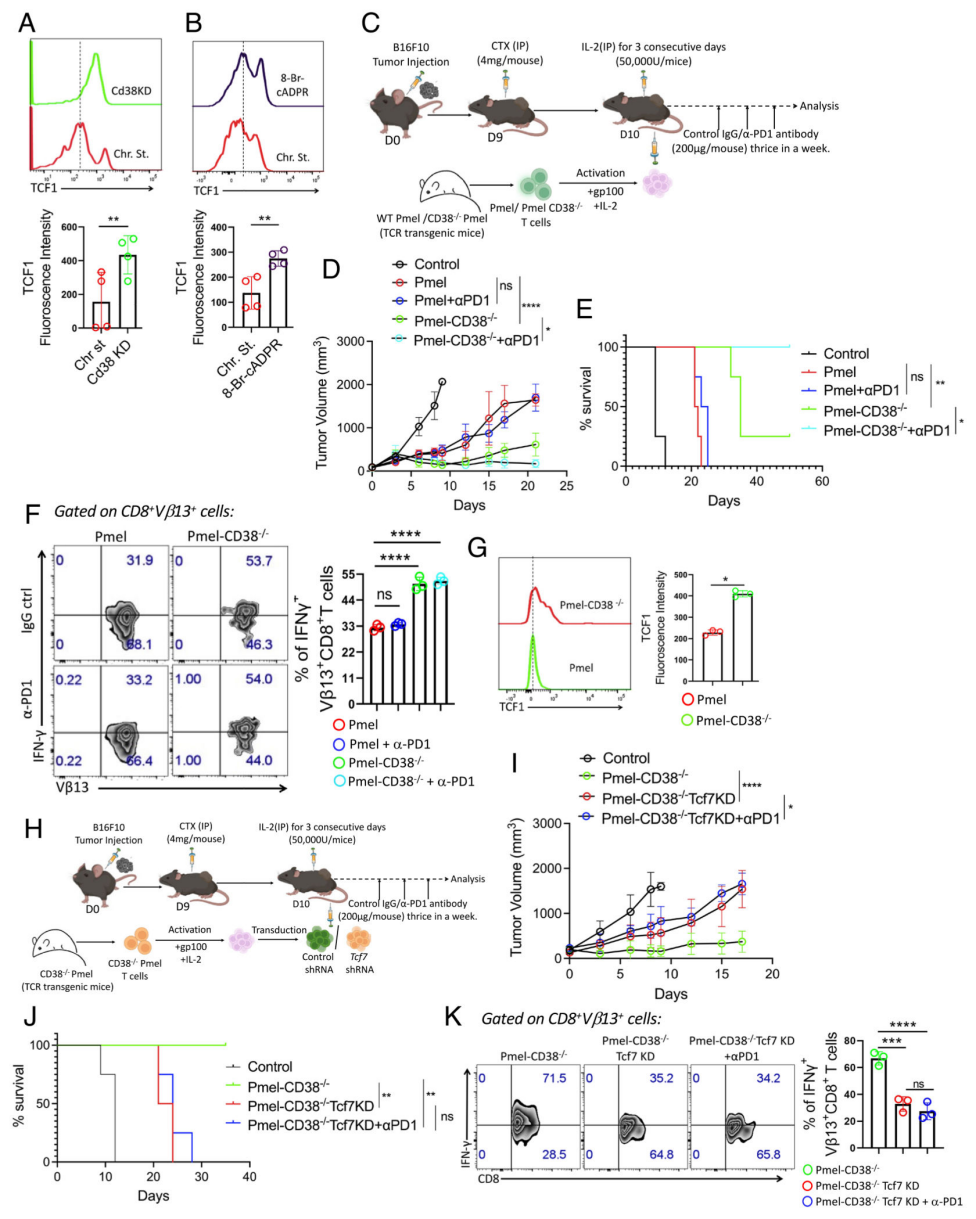
Inhibition of CD38 restores TCF1 expression and responsiveness to anti-PD1 therapy. (*A* and *B*) TCF1 expression in chronically expanded CD8^+^ T cells: (*A*) transduced with either control shRNA or CD38 shRNA, and (*B*) treated with either vehicle control or 8-Br-cADPR. *Bottom* panels represent cumulative data of median fluorescence intensity from four independent experiments. (*C*) Schematic representation of the ACT protocol where C57BL/6 mice (n = 4 mice/group) with subcutaneously established B16-F10 tumor adoptively transferred with 0.75 × 10^6^ WT Pmel or CD38^−/−^ Pmel T cells with or without anti-PD1 treatment (200 μg/mouse, thrice a week) and were evaluated for: (*D*) tumor growth, and (*E*) survival. (*F* and *G*) Assessment of (*F*) intracellular cytokine production, and (*G*) TCF1 expression in CD8^+^Vβ13^+^ T cells obtained from the tumor site. (*H*) Schematic representation of the adoptive transfer strategy of Pmel-CD38^−/−^ and Pmel-CD38^−/−^ Tcf7KD T cells with or without anti-PD1 treatment in mice (n = 4) subcutaneously established B16-F10 melanoma tumor. (*I*) Mean tumor volume at different time points is presented. (*J*) KM curves for time-to-killing for experimental conditions are shown. (*K*) IFNγ production by intratumoral CD8^+^Vβ13^+^ T cells and the *Bottom* panel represent the cumulative data from three different mice. *P < 0.05; **P < 0.01; ***P < 0.005; ****P < 0.0001; ns, nonsignificant.

Given that the expression of CD38 is primarily associated with intratumoral Tex^term^ cells, we, next, sought to determine whether CD38 has any role in orchestrating terminal exhaustion in T cells. Chronically stimulated (11 d) human CD8^+^ T cells transduced with either control shRNA or CD38 shRNA (SI Appendix, Fig. S4*A*) were assessed both phenotypically and functionally for their exhaustion features. We observed that the PD1 and Tox levels were comparable between control shRNA and CD38 shRNA transduced cells, except for Tim3, which exhibited a significant decrease in CD38 knockdown cells (SI Appendix, Fig. S4 *B*–*D*). Most interestingly, we noted that shRNA-mediated knockdown of CD38 restored the loss of TCF1 expression in chronically stimulated human CD8^+^ T cells (Fig. 4*A*). Moreover, the knockdown of CD38 also ameliorated the functionality of Tex, as evident by their increased IFNγ and TNFα production (SI Appendix, Fig. S4*E*).

Since CD38 catalyzes the cyclization of NAD^+^ to cADPR, an important signaling mediator in various cell types (20, 28), we next elucidated the role of CD38-induced downstream signaling in regulating the terminal exhaustion in CD8^+^ T cells. We pharmacologically blocked CD38-induced signaling by using 8-Bromo-cADPR (8Br-cADPR), a stable, cell-permeable analogue of cADPR. Akin to CD38 knockdown, 8Br-cADPR treatment also had minimal effect on PD1 and Tox expression levels but downregulated Tim3 expression in chronically stimulated CD8^+^ T cells (SI Appendix, Fig. S4 *F*−*H*). Most importantly, blocking CD38 sustained the expression of TCF1 (Fig. 4*B*), as well as increased their nuclear localization in chronically stimulated CD8^+^ T cells (SI Appendix, Fig. S4*I*). Furthermore, we also noticed improved effector cytokine production by chronic CD8^+^ T cells upon 8Br-cADPR treatment (SI Appendix, Fig. S4*J*).

We next assessed whether genetic ablation of CD38 in tumor-reactive CD8^+^ T cells improved their anti-tumor response as well as responsiveness to anti-PD1 therapy. ACT using Pmel or CD38 knockout Pmel (Pmel-CD38^−/−^) T cells either alone or in combination with anti-PD1 therapy (Fig. 4*C*) delayed tumor progression in mice bearing B16-F10 mouse melanoma tumor as compared to mice without ACT (control) (Fig. 4 *D* and *E*). However, tumor control was transient, and no overt benefit upon anti-PD1 therapy was noted in mice receiving wild-type Pmel T cells. In contrast, Pmel-CD38^−/−^ T cells induced a robust anti-tumor response, which was further boosted upon anti-PD1 therapy (Fig. 4 *D* and *E*). Commensurate with improved anti-tumor potential, deficiency of CD38 substantially increased the functionality of adoptively transferred T cells (Vβ13^+^CD8^+^ T cells) at the tumor site compared to WT Pmel T cells (Fig. 4*F*). Interestingly, we also observed that Pmel-CD38^−/−^ cells retrieved from the tumor site exhibited high TCF1 expression compared to WT Pmel cells (Fig. 4*G*).

Finally, to ascertain the therapeutic superiority of Pmel-CD38^−/−^ T cells was in part due to the maintenance of TCF1 expression, we knockdown *Tcf7* in Pmel-CD38^−/−^ T cells using shRNA (termed as Pmel-CD38^−/−^ Tcf7KD) (SI Appendix, Fig. S4*K*), and adoptively transferred these cells in mice bearing B16-F10 melanoma tumor (Fig. 4*H*). The knockdown of *Tcf7* not only weakened the anti-tumor potential of Pmel-CD38^−/−^ T cells but also compromised their responsiveness to anti-PD1 therapy (Fig. 4 *I* and *J*). In line with these results, Pmel-CD38^−/−^ Tcf7KD cells showed a marked decrease in IFNγ production at the tumor site in contrast to Pmel-CD38^−/−^ T cells transduced with an empty vector (Fig. 4*K*). Notably, even though the data displayed in Fig. 4*K* had low event counts, significant differences were still obtained. These findings strongly indicate that acquiring CD38 expression on tumor-reactive CD8^+^ T cells leads to impaired anti-tumor potential and responsiveness to anti-PD1 therapy due to the loss of TCF1 expression.

### CD38 Triggers the Elevation of Intracellular Ca^2+^ Levels and Promotes Terminal Exhaustion in CD8^+^ T cells

**Fig. 5:**
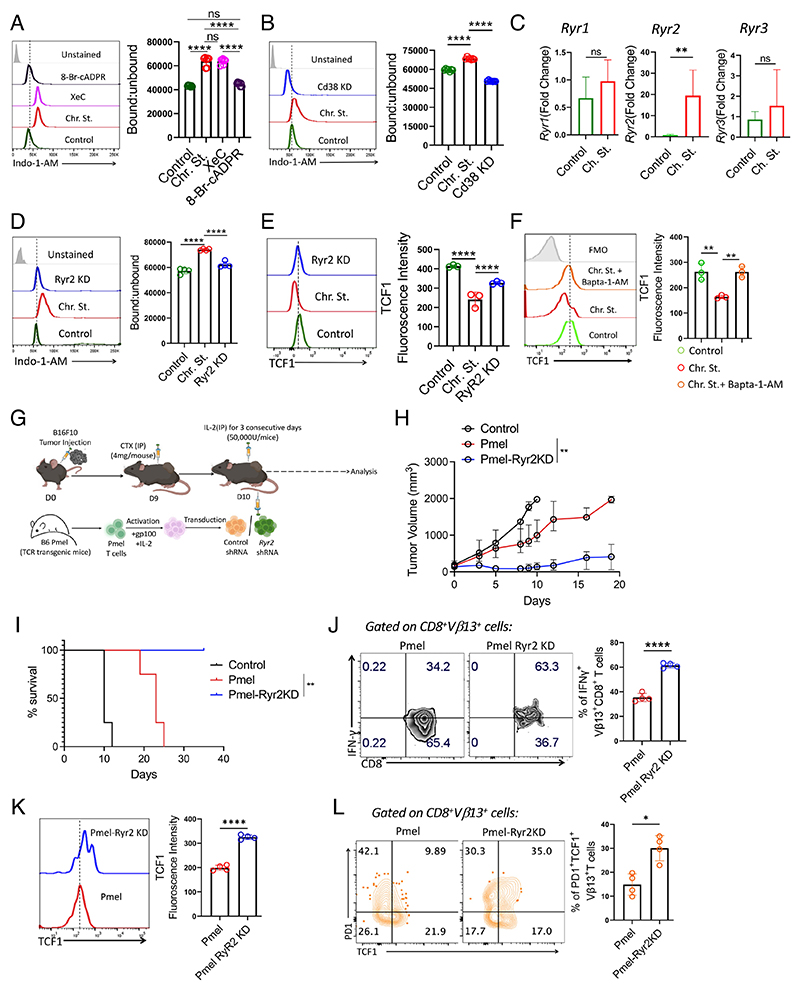
CD38 induces elevation of intracellular Ca^2+^ levels through the cADPR–RyR2 axis. (*A* and *B*) Intracellular calcium level as measured by ratio of bound to unbound Indo-1-AM in *A* control and chronically stimulated T cells treated either with XeC (5 µM) or 8-Br-cADPR (2.5 µM), (*B*) control and chronically stimulated T cells transduced either with control shRNA or CD38 shRNA. (*C*) Transcript levels of *Ryr1, Ryr2*, and *Ryr3*. (*D* and *E*) Control and chronically stimulated T cells transduced either with control shRNA or Ryr2 shRNA were assessed for (*D*) intracellular calcium level as measured by the ratio of bound to unbound Indo-1-AM and (*E*) expression of TCF1. (*F*) Control and chronic stimulated CD8^+^ T cells treated with or without Bapta-1-AM (13 µM) were evaluated for the expression of TCF1. (G) Schematic presentation of the experimental strategy and the differences observed in (*H*) tumor growth and (*I*) survival of tumor-bearing mice when subcutaneously established B16-F10 tumor in C57BL/6 mice (n = 4 mice/group) were treated by adoptively transferring 0.75 × 10^6^ Pmel T cells transduced with either control shRNA or Ryr2 shRNA. (*J* and *K*) Vβ13^+^CD8^+^ T cells retrieved from the tumor site were evaluated for (*J*) intracellular production of IFNγ, and (*K*) expression of TCF1. (*L*) Frequencies of tumor-derived Vβ13^+^CD8^+^ T cells expressing PD1 and TCF1. Adjacent bars represent cumulative data from (*A* and *B*) seven, (*C* and *D*) four, (*E* and *F*) cumulative data of mean fluorescence intensity from three, (*L*) frequency of PD1^+^TCF1^+^ T cells from four independent experiments. *P < 0.05; **P < 0.01; ***P < 0.005; ****P < 0.0001; ns, nonsignificant.

It is apparent from emerging evidence that exhausted T cells maintain high intercellular Ca^2+^ levels (5, 29). In line with this finding, we observed that chronically stimulated human CD8^+^ T cells exhibited elevated intracellular Ca^2+^ levels compared to CD8^+^ T cells expanded without chronic TCR stimulation (Fig. 5*A*). Regulation of intracellular Ca^2+^ levels in T cells is critically mediated through the release of Ca^2+^ from ER stores through inositol-1,4,5-trisphosphate (IP_3_) receptors (IP_3_R) and ryanodine receptors (RyRs) upon binding to IP_3_ and cADPR respectively (30, 31). Interestingly, we observed that high intracellular Ca^2+^ levels in chronically stimulated human CD8^+^ T cells were dependent on the CD38–cADPR axis as 8Br-cADPR treatment but not xesto-spongin C (XeC) treatment (inhibitor of IP3R mediated Ca^2+^ release from ER) significantly reduced the intracellular Ca^2+^ levels in chronically activated CD8^+^ T cells (Fig. 5*A*). Consistent with pharmacological inhibition, shRNA-mediated knockdown of CD38 also abated intracellular Ca^2+^ levels in chronic Tex cells (Fig. 5*B*).

Next, we explored the role of RyRs in mediating intracellular Ca^2+^ levels in chronic Tex cells. We first checked the transcript levels of different RyRs and noted a ~20-fold upregulation of only *Ryr2* expression in chronic Tex cells as compared to the control (Fig. 5*C*). However, *Ip3r* expression was comparable between the control and chronic stimulation groups (SI Appendix, Fig. S5*A*). Furthermore, shRNA-mediated knockdown of *Ryr2* (SI Appendix, Fig. S5*B*) blocked the elevation of intracellular Ca^2+^ levels in chronic Tex cells (Fig. 5*D*), suggesting that CD38 induced activation of the cADPR–RyR2 axis is important for the maintenance of high intracellular Ca^2+^ levels in Tex resulting from persistent antigen stimulation.

It has been shown that chronic Ca^2+^ signaling regulates various aspects of T cell exhaustion, while its inhibition keeps T cells in a less differentiated state (29). Since we observed that RyR2 was crucial in maintaining high intracellular Ca^2+^ levels in chronically stimulated CD8^+^ T cells, we sought to determine its role in promoting exhaustion in T cells. To this end, we knocked down *Ryr2* by shRNA in chronically expanded mouse CD8^+^ T cells and checked the expression of various exhaustion-associated markers. Surprisingly, shRNA-mediated knockdown of *Ryr2* had no effect on PD1 (SI Appendix, Fig. S5*C*) expression but significantly reduced CD38 expression (SI Appendix, Fig. S5*D*), indicating a feed-forward regulation between CD38 and RyR2-Ca^2+^ axis in Tex cells. Most intriguingly, we noted that knockdown of *Ryr2* substantially restored TCF1 expression in CD8^+^ T cells expanded in the presence of persistent antigen exposure (Fig. 5*E*). Similar restoration in TCF1 levels in chronic Tex cells was also observed upon chelation of intracellular Ca^2+^ using Bapta-1-AM (Fig. 5*F*), further suggesting the crucial role of RyR2-dependent Ca^2+^ in regulating TCF1 expression in Tex cells. Moreover, *Ryr2* knock-down also ameliorated intracellular cytokine production by chronic Tex cells (SI Appendix, Fig. S5*E*). These data together indicate that terminal differentiation of CD8^+^ T cells resulting from chronic TCR stimulation can partly be rescued by blocking RyR2-mediated elevation of intracellular Ca^2+^ levels.

Considering the alleviation of T cell exhaustion upon genetic knockdown of *Ryr2*, we next checked whether inhibiting it would improve the anti-tumor potential of T cells. We adoptively transferred Pmel T cells transduced with either control shRNA or *Ryr2* shRNA (referred to as Pmel-Ryr2KD cells) into mice bearing B16-F10 melanoma tumor (Fig. 5*G*). Compared to control shRNA-transduced Pmel T cells, Pmel-Ryr2KD cells mounted a robust anti-tumor response and prolonged the survival of mice (Fig. 5 *H* and *I*). Importantly, Pmel-Ryr2KD cells obtained from the tumor site exhibited improved functionality (Fig. 5*J*) and retention of TCF1 expression (Fig. 5*K*) compared to wild-type Pmel T cells. Despite the low event counts in the results shown in Fig. 5*J*, significant differences were noted. Interestingly, compared to wild-type Pmel T cells, tumor-derived Pmel-Ryr2KD cells also exhibited a significant expansion of progenitor or memory-like CD8^+^ T cells, identified as PD1^+^TCF1^+^ T cells (Fig. 5*L*), previously shown to mediate durable tumor control (13). Thus, CD38 induced terminal exhaustion in CD8^+^ T cells, at least in part attributed to the cADPR–RyR2 axis–mediated maintenance of elevated intracellular Ca^2+^ levels, which hinder the differentiation of progenitor CD8^+^ T cells.

### CD38–RyR2 Axis–Induced Ca^2+^ Levels Block TCF1 Expression in Chronically Stimulated CD8^+^ T cells by Promoting AKT Activation

**Fig. 6:**
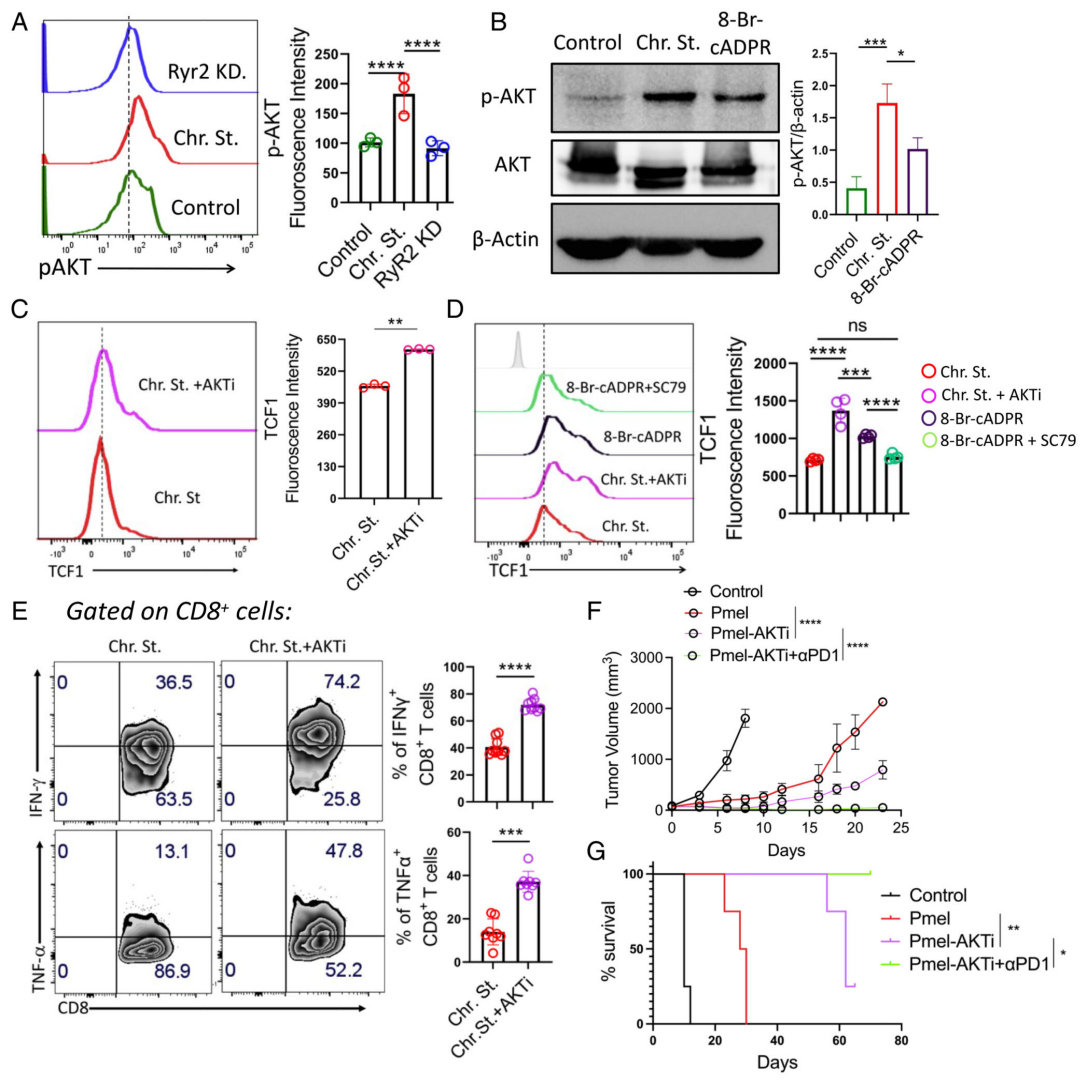
CD38–RyR2 axis, by promoting AKT activation, impedes TCF1 expression in exhausted T cells. (*A* and *B*) Expression of p-AKT level (Ser^473^) in (*A*) control and chronically stimulated T cells transduced with either control shRNA or Ryr2 shRNA by flow cytometry and (*B*) control and chronically stimulated T cells treated with either vehicle control or 8-Br-cADPR by western blot. (*C* and *D*) TCF1 expression in (*C*) chronically stimulated T cells treated with or without AKTi and (*D*) chronically stimulated T cells treated with either AKTi or 8-Br-cADPR or 8-Br-cADPR+SC-79. (*E*) Chronically stimulated CD8^+^ T cells expanded in the presence or absence of AKTi were checked for their cytokine production by flow cytometry. (*F* and *G*) C57BL/6 mice (n = 4/group) bearing B16-F10 tumor were either kept untreated or adoptively transferred with 0.75 × 10^6^ Pmel T cells activated in the presence or absence of AKTi. Groups of mice receiving Pmel-AKTi T cells were either administered with control IgG or Anti-PD1 antibody (200 μg/mouse, thrice per week). (*F*) Data in the figure demonstrate the mean tumor volume at different time points. (*G*) KM curves for time-to-killing for experimental conditions are shown. Data are representative of (*A*) three (cumulative data of mean fluorescence intensity), (*B*) three, (*C*) three (cumulative data of mean fluorescence intensity), (*D*) four (cumulative data of median fluorescence intensity, and (*E*) eight independent experiments. *P < 0.05; **P < 0.01; ***P < 0.005; ****P < 0.0001; ns, nonsignificant.

Although dynamic changes in intracellular Ca^2+^ concentrations are crucial for T cell activation, differentiation, and effector function, chronic Ca^2+^ levels have been implicated in inducing T cell dysfunction (29, 31). So, we wanted to delineate the signaling pathway triggered by chronic Ca^2+^ levels in promoting terminal exhaustion in CD8^+^ T cells. Intracellular Ca^2+^ can regulate numerous signaling pathways, including the activity of the PI3K–AKT pathway, which has been shown to be associated with T cell dysfunction in tumor (32, 33). In fact, GO analysis of chronic Tex cells also demonstrated the enrichment of the PI3K–AKT pathway as one of the top 20 significant pathways in cells present in cluster 4, which exhibited terminal exhaustion (SI Appendix, Fig. S2*F*). So, we sought to determine whether RyR2-mediated elevation of intracellular Ca^2+^ in chronically Tex led to the sustained activation of AKT, subsequently promoting the differentiation of terminally differentiated CD8^+^ T cells. We assessed AKT activation (as measured by the phosphorylation at Ser^473^) and observed augmented AKT phosphorylation (p-AKT) in chronically stimulated CD8^+^ T cells, which was significantly downregulated upon genetic knockdown of *Ryr2* (Fig. 6*A*). Next, to ascertain the role of CD38 in triggering RyR2-dependent activation of AKT, CD38 was inhibited both pharmacologically (using 8-Br-cADPR) and shRNA-mediated knockdown, and p-AKT levels were assessed. Both 8-Br-cADPR treatment and *Cd38* knockdown led to the downregulation of p-AKT levels in chronically stimulated CD8^+^ T cells (Fig. 6*B* and SI Appendix, Fig. S5*F*), further establishing the fact that the CD38–RyR2 axis maintained AKT activation in Tex.

Next, we wanted to determine whether inhibition of AKT has any role in regulating TCF1 expression and hence determines Tex^term^ cell fate. Interestingly, inhibition of AKT using AKTi during the chronic expansion of CD8^+^ T cells preserved TCF1 expression (Fig. 6*C*). In contrast, stabilization of AKT activation using a small molecule agonist, SC79, abrogated the effect of CD38 inhibition-mediated maintenance of TCF1 expression in chronically stimulated CD8^+^ T cells (Fig. 6*D*). The data together point to the fact that activation of AKT impedes the maintenance of TCF1 expression and promotes the differentiation of Tex^term^ cells. Moreover, inhibition of AKT also improved the functionality of chronically stimulated CD8^+^ T cells (Fig. 6*E*). However, it had no influence in regulating the expression of immune checkpoint molecules, except for CD38 expression, which was markedly down-regulated (SI Appendix, Fig. S5 *G*–*I*). Further, to assess whether AKT inhibition endows CD8^+^ T cells with superior anti-tumor potential and improves their responsiveness to anti-PD1 therapy, vehicle control or AKTi treated Pmel CD8^+^ T cells were adoptively transferred to mice baring B16-F10 melanoma (SI Appendix, Fig. S5*J*). Pharmacological inhibition of AKT not only improved the anti-tumor potential of Pmel CD8^+^ T cells and prolonged the survival of the mice but also rendered the Pmel CD8^+^ T cells responsive to anti-PD1 therapy (Fig. 6 *F* and *G*).

## Discussion

T cells under persistent antigen exposure, such as chronic viral infection and cancer, progressively transition into a terminally differentiated state, rendering them hyporesponsive to anti-PD1 therapy (26, 34, 35). In the present study, we set out experiments to delineate the molecular mechanism underlying the loss of responsiveness of Tex to ICB therapy. Our findings reveal that the sustained expression of CD38 on Tex triggers an intracellular Ca^2+^-dependent signaling pathway through activation of the cADPR–RyR2 axis. This pathway plays a significant role in the loss of TCF1 expression, subsequently rendering Tex cells resistant to immunotherapy.

CD38, a multifunctional transmembrane protein, was initially recognized as a marker for T cell activation and maturation (36). However, recent studies have identified the multifaceted role of CD38, placing it as an important molecular rheostat controlling a diverse array of T cell functionality (21, 37). Expression of CD38 has also been reported in intratumoral T cells exhibiting impaired anti-tumor response (21). Moreover, CD8^+^ T cells co-expressing PD1 and CD38 (PD1^+^CD38^hi^CD8^+^ T cells) have been identified as a dysfunctional T cell subset that inversely correlates with the therapeutic responsiveness of anti-PD1 therapy in patients with metastatic melanoma (17). These studies have led us to comprehensively characterize the phenotypic, functional, and molecular traits of CD38-expressing intratumoral CD8^+^ T cells and precisely understand their role, if any, in regulating the responsiveness of T cells to ICB therapy. Our data suggest that Tex cells can be stratified into two subsets based on CD38 expression: CD38^lo^ and CD38^hi^, with the latter representing the Tex^term^ cells due to their co-expression of PD1 and Tim3, elevated Tox levels, and functional deficits. This finding aligns with previous reports placing CD38 in intratumoral T cell subsets that display terminal differentiation and incapability for functional rejuvenation (9, 17, 18). Consistently, analysis of scRNA-seq data from metastatic melanoma patients treated with either anti-PD1 or anti-PD1+anti-CTLA4 (9), highlighted the increased abundance of CD38-expressing CD8^+^ T cells in patients non-responding to ICB therapy, as previously reported (17). Most interestingly, we noted an inverse correlation between *CD38* expression and *TCF7* levels, suggesting that CD38 expression on Tex cells could, in part, contribute to the nonresponsiveness to anti-PD1 therapy, possibly by interfering with TCF1 levels. Indeed, we found that CD38 expression negatively regulated TCF1 expression in CD8^+^ T cells expanded with chronic TCR stimulation. This regulation was pivotal in determining the responsiveness to ICB therapy, as tumor-reactive CD8^+^ T cells with genetic ablation of CD38 (Pmel-CD38^−/−^ T cells) exhibited a robust anti-tumor response to anti-PD1 therapy; however, this effect was abrogated upon *Tcf7* knockdown. Therefore, our data provided a mechanistic understanding of the recently published work demonstrating the failure of anti-PD1 immunotherapy in patients with an increased intratumoral abundance of PD1^+^CD38^+^CD8^+^ T cells (17). Congruently, we also observed that the dysfunctionality of Tex cells, in terms of their cytokine production, could partially be rescued by blocking CD38, suggesting the role of CD38 in regulating the functional fate of the T cells (17). The role of CD38 in regulating effector cytokine production by Tex has also been demonstrated in mice with chronic LCMV infection (38). However, the study, in contrast to our findings, showed that CD38 deletion in LCMV-specific CD8^+^ T cells resulted in a modest but significant decrease in the frequency of TCF1^+^ Tpex cells during chronic LCMV Cl13 infection (38). We propose that the reduced frequency of TCF1^+^P14^+^ T cells upon CD38 deletion may not be attributed to the decreased expression of TCF1 but instead to increased proliferation and subsequent differentiation of TCF1^+^ Tpex cells into highly effector TCF1^-^PD1^+^ Tex cells, as observed earlier in the case of chronic LCMV infection (12, 38, 39). Therefore, it is possible that CD38^−/−^ LCMV-specific P14^+^ T cells, compared to wild-type P14^+^ T cells, had high TCF1 expression on a per-cell basis, triggering their massive expansion and differentiation into the effector subset upon antigen encounter in mice, while maintaining a small pool of TCF1^+^P14^+^ T cells (12, 39). Nevertheless, further research is essential to define whether CD38 has any context-specific role in influencing the differentiation of exhausted CD8^+^ T cell subsets, particularly in chronic LCMV infection vs. tumor-bearing hosts.

The most compelling role of CD38, we identified, in regulating T cell exhaustion was sustained maintenance of intracellular Ca^2+^ levels through the cADPR–RyR2 axis. CD38 is a cell surface protein with both NADase and ADP-ribosyl cyclase activity, which utilizes cellular NAD^+^ to generate cADPR (37, 40). It has been shown that cADPR, an important intracellular second messenger, can significantly and specifically stimulate RyR-mediated Ca^2+^ release from the ER lumen (41, 42). Herein, in chronically Tex, RyR2 appeared to be crucial in the mobilization of Ca^2+^ from ER and hence maintained elevated intracellular Ca^2+^ levels. Recently, the role of store-operated calcium entry (SOCE) in maintaining elevated intracellular Ca^2+^ levels has been implicated in promoting CAR-T cell dysfunction (29). Although we haven't explored the role of SOCE in T cell exhaustion but argued that RyR2 and SOCE might act in concert in maintaining intracellular Ca^2+^ levels, as RyR2-mediated depletion of ER Ca^2+^ levels has been shown to trigger Ca^2+^ entry through SOCE (31, 43).

Hyperactivated intracellular Ca^2+^ levels have been found to be associated with T cell dysfunctionality and diminished ability to control tumor growth (29). In fact, the ineffectual anti-tumor response displayed by CAR-T cells expanded with tonic signaling has been primarily attributed to the Ca^2+^-dependent activation of the NFAT pathway (29, 44). Herein, we observed that elevated intracellular Ca^2+^ levels in chronically Tex were responsible for the chronic activation of AKT. The Ca^2+^-dependent activation of AKT can be mediated in at least two independent ways: a) Ca^2+^ can directly activate PI3K, leading to increased PIPâ,*f* production and subsequent AKT activation, or b) calcium can regulate the activity of phosphatases and kinases involved in the PI3K–AKT pathway, thus influencing the phosphorylation of AKT (45–47). Never-theless, prolonged AKT signaling was not only found to impinge on the effector function of the T cells but, importantly, hindered the TCF1 expression and, hence the responsiveness of exhausted T cells to anti-PD1 therapy.

In conclusion, our study underscores the importance of CD38 as a key player in suppressing TCF1 expression and the subsequent refractoriness of exhausted T cells to anti-PD1 therapy. These findings emphasize the significance of CD38 as a potential target for therapeutic intervention, aiming to enhance the effectiveness of ICB therapy in individuals with malignancies.

## Materials and Methods

### Chronic Stimulation of Human CD8^^+^^ T cells

PBMCs were isolated by Ficoll-hypaque gradient centrifugation from buffy coats of healthy human subjects. Buffy coats were de-identified prior to use in the study, and all samples were used in accordance with Institutional IRB approval (No. IICB/IRB/2020/2P). Magnetic bead purified CD3/CD8 T cells were activated for 72 h with plate-bound anti-CD3 (5 µg/mL) and anti-CD28 (2 µg/mL), along with recombinant human IL-2 (100 units/mL). Subsequently, the T cells were either expanded with chronic stimulation (plate-bound anti-CD3 at 2 μg/mL) or without stimulation (only IL-12 at 100 units/mL) for an additional 8 d before being utilized for data acquisition. Detailed protocols can be found in SI Appendix.

## ACT Protocol

B16-F10 melanoma (0.5 × 106 cells) were subcutaneously injected into 8 to 10-wk-old C57BL/6 mice. Nine days post-tumor engraftment, lympho-depletion was done with cyclophosphamide mono-hydrate (4 mg/mouse). The next day, tumor-bearing mice were either left untreated or adoptively transferred (i.v.) with three days of activated Pmel T cells or Pmel-CD38^−/−^ T cells or control shRNA/target shRNA transduced Pmel T cells at 0.75 × 10^6^ cells/mouse. IL2 (50,000 U/mouse; i.p.) was administered to recipient mice for three consecutive days post-ACT. In some cases, post-ACT, mice received control IgG or anti-PD1 antibody (200 μg/mouse) thrice a week.

### Single Cell Sequencing

To perform a whole transcriptomics analysis of single cells, we used microwell-seq barcoding technology (BD Rhapsody platform, BD Biosciences). For single-cell capture and cDNA synthesis with the BD Rhapsody express single-cell analysis system, 20,000 cells with >90% viability were loaded onto primed microwells of BD Rhapsody cartridge followed by the addition of beads with oligonucleotide barcoded to saturation as per the manufacturer’s protocol. Detailed protocols and data analysis can be found in SI Appendix.

### Data, Materials, and Software Availability

scRNA sequencing files data have been deposited in NCBI GEO (GSE251829) (48). All other data are included in the article and/or SI Appendix.

## Supplementary Material

This article contains supporting information online at https://www.pnas.org/lookup/suppl/doi:10.1073/pnas.2315989121/-/DCSupplemental.

Figures S1 to S5

## Significance

The success of immunotherapy relies on understanding the resistance mechanisms that impede the ability of T cells to respond to therapy. Phenotypic, functional, and single-cell transcriptomic analysis of intratumoral CD8^+^ T cells from both immunotherapy-resistant pre-clinical models and metastatic melanoma patients identifies that CD38-expressing CD8^+^ T cells are associated with anti-PD1 resistance. Exploring the underlying molecular mechanism reveals the crucial role of the CD38-induced signaling cascade in contributing to the development of immunotherapy resistance by directly suppressing TCF1 expression. Collectively, our data highlight CD38 as an important immune checkpoint that impedes the responsiveness of CD8^+^ T cells to immunotherapy. Consequently, it offers a rationale for targeting CD38 to enhance the therapeutic efficacy of PD1 blockade therapy in cancer.
