# Supplementary material for: CD38–RyR2 axis–mediated signaling impedes CD8+ T cell response to anti-PD1 therapy in cancer
Source: Proc Natl Acad Sci U S A. Author manuscript; Available in PMC 2024 Mar 19. (PMC10945783; doi:10.1073/pnas.2315989121)
Supplement: Figures S1 to S5 [file EMS194613-supplement-Figures_S1_to_S5.pdf]

Supporting Information for

CD38-RyR2 axis mediated signaling impedes $CD8^+$ T cell response to anti-PD1 therapy in cancer

Anwesha Kar[1,8], Puspendu Ghosh[1], Anupam Gautam[2,3#], Snehanshu Chowdhury[1,8#], Debashree Basak[1,8], Ishita Sarkar[1,8], Arpita Bhoumik[1], Shubhrajit Barman[4,8], Paramita Chakraborty[5], Asima Mukhopadhyay[6], Shikhar Mehrotra[5], Senthil Kumar Ganesan[4], Sandip Paul[7], Shilpak Chatterjee[1*]

Corresponding author: Shilpak Chatterjee
Email: schatterjee@iicb.res.in

This PDF file includes:

Supporting text
Figures S1 to S5
Tables S1
Legends for Figures S1 to S5
SI References

Supporting Information Text

Materials and Methods

Mice

C57BL/6 strain were specifically bred within the animal facility of the CSIR-Indian Institute of Chemical Biology (CSIR-IICB). Wild-type Pmel mice were obtained from Jackson Laboratory (Bar Harbor, MA), while Pmel-CD38$^{-/-}$ mice were generated by Dr. Shikhar Mehrotra's lab at the Medical University of South Carolina, USA. The animals were housed in pathogen-free facilities, and all experimental protocols received approval from the Institutional Animal Ethics Committee of CSIR-IICB, Kolkata, India. In tumor control experiments, an equal number of mice of 6-8 weeks of age, matched for gender (including both males and females), were randomly selected for experimentation. It was observed that the outcomes of the studies were not influenced by the sex of the mice.

Cell lines

The B16-F10, HEK293T, and YUMM1.7 cell lines were procured from the ATCC. To ensure their *Mycoplasma* contamination status, all cell lines were subjected to Mycoplasma testing using the MycoAlert Mycoplasma Detection Kit (Lonza), and they tested negative. For experiments involving injection into mice or transfection with plasmids for lentivirus production, cells within the 4 to 6 passage range were utilized.

Chronic stimulation of human CD8$^+$ T cells

Peripheral blood mononuclear cells (PBMCs) were isolated via Ficoll-hypaque gradient centrifugation from de-identified buffy coats obtained from healthy human subjects. Naïve CD3/CD8 T cells were purified using the Dynabeads Untouched Human CD3$^+$ T-cell isolation kit or Dynabeads Untouched Human CD8$^+$ T-cell isolation kit (Invitrogen). Purified CD3/CD8 T cells were activated with plate-bound anti-CD3 (5 µg/ml) and anti-CD28 (2 µg/ml), along with recombinant human IL-2 (100 units/ml), in complete RPMI-1640 supplemented with 10% FBS (Invitrogen) for 72 hours. Following 72 h of activation, T cells were passaged at every 48h at 1×10^6 cells/ml in RPMI-1640 medium containing 10% FBS, with (chronic) or without (referred to as control in the text) plate-bound anti-CD3 (2 µg/ml) until 11 days. The chronic group was maintained in recombinant human IL-2 (25 units/ml), while the control group was maintained in recombinant human IL-2 (100 units/ml). All the groups (both control and chronic) were stimulated overnight with anti-CD3 (2 µg/ml) before being used for taking readouts. 8-Bromo-cADPR was solubilized in DMSO/water (1:1). Xestospongin C (XeC), AKT Inhibitor VIII (AKTi) and SC-79 were dissolved in DMSO. The compounds 8-Bromo-cADPR (2.5 µM), XeC (5 µM), AKTi (1 µM), and SC-79 (0.5 µg/ml) were introduced after 72 hours of T cell activation and maintained throughout the experimental period.

Isolation of Tumor Infiltrating T Cells

To isolate tumor-infiltrating T cells (TILs) from mice bearing subcutaneously established B16-F10 melanoma and YUMM1.7 melanoma, tumors were excised and finely chopped using tweezers and

scissors. The chopped tumors were then subjected to digestion with 2 mg/ml collagenase type IV (Stemcell Technologies, Vancouver, BC) for 50 minutes. After digestion, the tumors were filtered through 70 µm cell strainers (BD Biosciences, San Jose, CA) and layered over Hi-Sep LSM (HiMedia Laboratories, India). Following centrifugation at 1200 rpm for 30 minutes, a layer of mononuclear cells containing TILs was separated and isolated. This cell layer underwent two washes and then dissolved in RPMI-1640 for subsequent staining procedures.

Flow Cytometry

Staining of cells for flow cytometry analysis was done following the protocol published earlier (1). Briefly, Cell surface markers were stained by incubating cells with specific fluorochrome-conjugated antibodies diluted in FACS buffer (0.5% BSA, 0.1% Sodium azide in PBS) for 30 minutes at 4°C. After incubation, cells were washed twice with PBS, resuspended in FACS buffer containing 1% paraformaldehyde, and acquired on the BD LSR Fortessa II (BD Biosciences, San Jose, CA).

For intracellular cytokine staining (IFNγ and TNFα), T cells were restimulated for 4 hours at 37°C with PMA (100 ng/ml), ionomycin (1 µg/ml), and Golgi Plug (BD Biosciences, San Jose, CA) before surface marker staining. After surface staining, cells were fixed and permeabilized using BD Cytofix/Cytoperm Kit (BD Biosciences, San Jose, CA) following the manufacturer's protocol and subsequently used for intracellular staining.

For staining of TCF1, Tox, and Ki67, cells were initially stained with surface markers and then fixed/permeabilized using a FoxP3 staining buffer set (Thermo Fisher Scientific, Waltham, MA). Fixed/permeabilized cells were then used for the staining of nuclear proteins.

For apoptosis staining, cells were initially incubated with fluorochrome-conjugated antibodies against surface markers, followed by Annexin V staining using the FITC Annexin V Apoptosis Detection Kit (BD Biosciences, San Jose, CA).

For all samples (except for those used for apoptosis analysis), after surface marker staining, cells were incubated with fixable live/dead staining dye (Live/Dead fixable yellow dead cell stain kit from Thermo Fisher Scientific, Waltham, MA) before being used for any downstream analysis. All the fluorochrome-conjugated antibody-stained samples were acquired on the BD LSR Fortessa II (BD Biosciences, San Jose, CA) and analyzed using FlowJo software (BD Biosciences, San Jose, CA).

Metabolic Flux analysis

The Seahorse XFe24 analyzer from Agilent Technologies was employed to determine the oxygen consumption rate (OCR), as shown earlier (1). In brief, human $CD8^+$ T cells (0.5×10^6 cells/well) were plated on a Seahorse culture plate coated with Cell-Tak (Corning, NY, USA), allowing for adherence for 30 minutes at 37^0C in a CO_2-free incubator. Subsequently, OCR analysis was performed under baseline conditions and in response to particular compounds: oligomycin (Oligo, 1 mmol/L), fluoro-carbonyl cyanide phenylhydrazone (FCCP, 1 mmol/L), and a combination of rotenone plus antimycin A (Rot-Anti A, 2 mmol/L and 100 nmol/L, respectively).

Immunoblotting

For p-AKT and total AKT evaluation, cells were lysed in RIPA buffer with a protease inhibitor cocktail (Thermo Fisher Scientific, Waltham, MA), and 30 µg of total protein was separated by 10% SDS-PAGE. After the transfer of the proteins to nitrocellulose membranes, primary antibody (Cell Signaling Technologies, Danvers, MA) incubation was carried out overnight at 4°C, followed by secondary antibody incubation (horseradish peroxidase–conjugated mouse anti-rabbit IgG, 1:2000 dilution, Jackson ImmunoResearch Laboratories, West Grove, PA) for 2 hours at room temperature. Concurrently, pre-stained protein markers (Invitrogen, Thermo Fisher Scientific, Waltham, MA) were run in parallel to identify the molecular weight of the detected proteins. Chemiluminescent detection with Clarity Western ECL substrate (Bio-Rad, Hercules, CA) was visualized on Bio-Rad Versadoc Imaging System. The same membrane was then stripped and re-probed with β-actin antibody (Cell Signaling Technologies, Danvers, MA) for normalization.

Confocal Microscopy

For confocal imaging, control and chronically expanded $CD8^+$ T cells were fixed (4% PFA), permeabilized (0.1% Triton-X solution), and blocked (1% BSA solution). Staining with rabbit anti-TCF1 antibody (Cell Signaling Technologies, Danvers, MA) was carried out overnight at 4°C on rotation. Subsequent steps included washing and staining with Alexa Fluor 647 conjugated anti-rabbit IgG (Jackson ImmunoResearch Laboratories, West Grove, PA) for 2 hours at room temperature with gentle rocking, and incubation with DAPI for 5 minutes. Following three washes, cells were pelletized on a glass slide using a cytospin centrifuge, mounted with Prolong Gold Anti-fade mounting media (Thermo Fisher Scientific, Waltham, MA), and cured overnight in the dark at room temperature. Images were acquired the following day using a Zeiss LSM980 Confocal Laser Scanning Microscope.

For visualizing mitochondria, T cells were fixed on glass bottom confocal dishes using Fibronectin (Sigma-Aldrich, St. Louis, MO) coated plates and stained with MitoTracker Green and DAPI. The images were acquired immediately on Zeiss LSM980 Confocal Laser Scanning Microscope.

qPCR analysis

For quantitative RT-PCR (qPCR), 1-2 × 10^6 cells were lysed using TRIzol reagent (Invitrogen, Thermo Fisher Scientific, Waltham, MA), and RNA was isolated following the manufacturer's protocol. The isolated RNA was resuspended in 25-30 µl UltraPure water (Invitrogen, Thermo Fisher Scientific, Waltham, MA), and quantified using a Multiskan SkyHigh Microplate Spectrophotometer(Invitrogen, Thermo Fisher Scientific, Waltham, MA). cDNA was synthesized from 1 µg of isolated RNA using either the iScript cDNA Synthesis kit (Bio-Rad, Hercules, CA) or the Super Reverse Transcriptase MuLVEasy Kit (BioBhara@ LifeScience Pvt. Ltd., Kolkata, India), according to manufacturer's protocol on T100 thermal cycler (Bio-Rad, Hercules, CA) and the resulting cDNA was diluted 1:3 in nuclease-free water for qPCR reactions using iTaq Universal SYBR Green Supermix (Bio-Rad, Hercules, CA)at standard reaction speed on a CFX96 Real-Time System (Bio-Rad, Hercules, CA) for 40 cycles.

Table S1. Primer sequences:

Human qPCR Primers: 5'-3'		
**Gene**	**Forward Primer**	**Reverse Primer**
Ryr1	CACCAATGGCCTATACAACCAG	GCTCAGGATAACGCCCTCG
Ryr2	GGCAGCCCAAGGGTATCTC	ACACAGCGCCACCTTCATAAT
Ryr3	GCACTCTTTCAGCGGAATGTA	GCAGGATGTATAGTCCACCAAC
Ip3r	CCACAGACGCAGTGCTACTC	GTCCCCAGCAATTTCCTGTTT
Cd38	AGACTGCCAAAGTGTATGGGA	GCAAGGTACGGTCTGAGTTCC
β-actin	CATGTACGTTGCTATCCAGGC	CTCCTTAATGTCACGCACGAT
**Mouse qPCR Primers: 5'-3'**		
Ryr2	ATGGCTTTAAGGCACAGCG	CAGAGCCCGAATCATCCAGC
Tcf7	AGCTTTCTCCACTCTACGAACA	AATCCAGAGAGATCGGGGGTC
β-actin	ACGTAGCCATCCAGGCTGGTG	TGGCGTGAGGGAGAGCAT

Single Cell sequencing

Capture of Single-cell and library preparation

The microwell-seq barcoding technology (BD Rhapsody platform) was utilized for single cell transcriptomics analysis (2, 3). Cell viability was determined using trypan blue on the ThermoFisher Scientific, Countess® II FL Automated Cell Counter. Approximately, $0.1X10^6$ T cells were labelled using BD™ Single-Cell Multiplexing Kit-Human with unique tags for each sample and BD™ AbSeq Ab-Oligos as per the manufacturer's guideline (Doc ID: 214419 Rev. 2.0). Subsequently, 20,000 cells (>90% viability) were loaded onto primed microwells of BD Rhapsody cartridge on BD Rhapsody express single-cell analysis system and oligonucleotide barcoded beads were added as per manufacturer's instructions (Doc ID: 210967 Rev. 1.0). Following cell and bead pairing in the microwells, cells were lysed and cDNA was prepared by reverse transcription from poly-adenylated RNA, captured on the beads. Finally, all sequencing libraries (Whole Transcriptome Analysis (WTA), Ab-Seq, and Sample Tag) were prepared using BD Rhapsody™ WTA Amplification kit as per the manufacturer's guideline (Doc ID: 23-21752-00).

Single-cell sequencing

Each indexed libraries were quantified using Qubit HS Assay (Invitrogen, Cat# Q32854). The obtained libraries were pooled and diluted to the final optimal loading concentration (1nm). The

pooled libraries were then denatured using 0.2N NaOH, spiked with 30% PhiX, and loaded onto Illumina Novaseq 6000 instrument to generate 150bp paired-end reads. The sequenced reads were available as BCL files and processed for data analysis. BCL files were then demultiplexed and converted to FASTQ format using the bcl2fastq tool. The required files were extracted from the FASTQ files using the Seven Bridges platform for further downstream analysis.

Single-cell sequencing data analyses

In the context of scRNA-seq analysis, the gene expression data was processed using the Seurat R package (v4.3.0) (4). The first step involved importing the processed gene expression matrix for each sample into Seurat, ensuring that all cells from each sample were retained. Quality control measures were applied to the data by filtering out cells with high mitochondrial gene expression and low feature counts, aiming to maintain data quality. Following quality control, the gene expression matrix of each sample was normalized using the "LogNormalize" method. This method scales the feature expression measurements for each cell by the total expression and applies a natural log transformation to the results. The normalized expression matrix was then used to identify the 2,000 most variable genes for each sample using the 'FindVariableFeatures' function with the 'vst' method. To facilitate further analysis, the gene expression matrices from all samples were merged into a single matrix. A linear transformation was applied to the merged matrix using the 'ScaleData' function, which is a standard pre-processing step prior to dimensional reduction techniques such as Principal Component Analysis (PCA). PCA was performed on the variable genes using the 'RunPCA' function in Seurat, capturing the main sources of variation in the dataset. To address potential batch effects, the scaled PCA matrix was processed with the 'RunHarmony' function (v1.0) (5) in Seurat. This function integrated the data into a shared space, reducing the impact of technical variations and allowing for unsupervised clustering. To visualize the data in a two-dimensional space, UMAP and t-SNE plots were generated using the batch-effect-corrected Harmony embeddings. The plots provided a visual representation of the cell clusters within the dataset. Cluster analysis was performed by constructing a Shared Nearest Neighbor (SNN) graph using the Harmony-reduced data, followed by the application of the Louvain clustering algorithm. This approach grouped cells into distinct clusters based on their similarity in gene expression patterns. Marker genes for each cluster were identified using the Seurat function 'FindAllMarkers', which helped characterize and distinguish different cell types or states. Visualization of the marker genes was achieved through the creation of heatmaps and feature plots using customized R code with the ggplot2 package. These visualizations provided insights into the gene expression patterns associated with different cell clusters. In addition, gene ontology analysis was performed using the identified marker genes (p-value < 0.05) in Metascape v3.5. The results of the gene ontology analysis were plotted using the ggplot2 R package, aiding in the interpretation of biological processes and functions associated with the identified marker genes. Single-cell RNA sequencing

(scRNA seq) data have been deposited in the Gene Expression Omnibus (GEO). GEO accession number GSE251829.

scRNASeq analysis of CD8^{+} T cells from metastatic melanoma bearing patient

We adopted the single-cell analysis methodology outlined by Trefny et al. (6) to reanalyze the intratumoral immune cell data from Sade-Feldman et al. This data was obtained from the GEO database under accession number GSE120575. Using the SingleCellExperiment (7) package in R, we created a single-cell object, followed by filtering, quality control, and normalization steps as described in the publication (6). Subsequently, CD8 T cells were defined and isolated from the other subtypes. The extracted subset from the single-cell object was then subjected to analysis (PCA, tSNE, and UMAP dimension reductions were computed). K-means was used for clustering with a specified number of centers (5, determined through iteration). The heatmap was generated using the plotHeatmap (8) function for the genes of interest. Spearman correlation was employed to assess correlations between genes, with a p-value of ≤ 0.05 considered statistically significant (visualized by corrplot (4). To determine the percentage of each cell type logcounts > 1 were retained as the threshold.

Lentiviral transduction of T cell

The transduction of T cells using lentiviral particles was done following the protocol published earlier (1). Briefly, HEK293T cells (7×10^6) were seeded in a T75 flask a day before transfection. Transfection included lentiviral plasmid DNA for target or control shRNA (Origene, Rockville, MD) with structural plasmids ps-PAX2 and p-MD2G, using the $CaCl_2$ and HBS method. After 24 hours, the medium was replaced with low serum-containing media. Four hours after media change, sodium butyrate was added at 5 mM concentration, and the cells were incubated for additional 48 to 72 hours, after which, lentivirus-containing supernatant was collected and filtered. The viral content was concentrated using LentiX Concentrator (Takara Bio, Shinga, Japan) following the manufacturer's protocol and stored at -80°C until use. Human CD8 T cells were activated for 72 h. After activation, cells were collected, washed, and transferred onto non-tissue culture–treated 24-well plates (Corning, NY, USA) coated overnight with Retronectin (30 µg/ml) (Takara Bio). Cells were transduced with concentrated *Cd38* shRNA lentiviral/control shRNA lentiviral supernatants, spinoculated at 1,000g for 2 hours at 32°C, and IL2 was added post-spinoculation, followed by a 48-hour incubation at 37°C. After the said time period, cells were collected and subjected to chronic stimulation for another six days and used for experimental readouts. For mouse T cells, splenocytes from wild-type Pmel or Pmel-CD38$^{-/-}$ mice were activated with gp100 peptide (1 µg/ml) and recombinant mouse IL-2 (100 units/ml) in complete RPMI with 10% FBS for 72 hours. After activation, cells were collected, washed, and transferred onto Retronectin-coated non-tissue culture–treated 24-well plates. Pmel-CD38$^{-/-}$ T cells were transduced with Tcf7 shRNA lentiviral/control shRNA lentiviral supernatants using the protocol mentioned above. In other sets of experiments, wild-type Pmel T cells or sorted CD8^{+} T cells from B6 mice were transduced with

Ryr2 shRNA lentiviral/control shRNA lentiviral supernatants. After 48 hours, cells were either adoptively transferred into mice or subjected to chronic antigen stimulation for up to 8 days before further analysis.

Statistical Analysis

All data presented, unless otherwise noted, is the mean (±SD) of three or five independent experiments. For statistical comparisons between two groups, the non-parametric, unpaired Wilcoxon-Mann-Whitney U test was utilised. In one-way or two-way ANOVA, comparisons involving more than two groups were employed. The programme GraphPad Prism 9 was used to analyse the data.

SI References

1. S. Chowdhury *et al.*, Intracellular Acetyl CoA Potentiates the Therapeutic Efficacy of Antitumor CD8+ T Cells. *Cancer Res* **82**, 2640-2655 (2022).
2. P. Chattopadhyay *et al.*, Single-cell multiomics revealed the dynamics of antigen presentation, immune response and T cell activation in the COVID-19 positive and recovered individuals. *Front Immunol* **13**, 1034159 (2022).
3. D. Thakral *et al.*, Integrated single-cell transcriptome analysis of CD34 + enriched leukemic stem cells revealed intra- and inter-patient transcriptional heterogeneity in pediatric acute myeloid leukemia. *Ann Hematol* **102**, 73-87 (2023).
4. T. Stuart *et al.*, Comprehensive Integration of Single-Cell Data. *Cell* **177**, 1888-1902 e1821 (2019).
5. I. Korsunsky *et al.*, Fast, sensitive and accurate integration of single-cell data with Harmony. *Nat Methods* **16**, 1289-1296 (2019).
6. M. P. Trefny *et al.*, Deletion of SNX9 alleviates CD8 T cell exhaustion for effective cellular cancer immunotherapy. *Nat Commun* **14**, 86 (2023).
7. R. A. Amezquita *et al.*, Orchestrating single-cell analysis with Bioconductor. *Nat Methods* **17**, 137-145 (2020).
8. D. J. McCarthy, K. R. Campbell, A. T. Lun, Q. F. Wills, Scater: pre-processing, quality control, normalization and visualization of single-cell RNA-seq data in R. *Bioinformatics* **33**, 1179-1186 (2017).

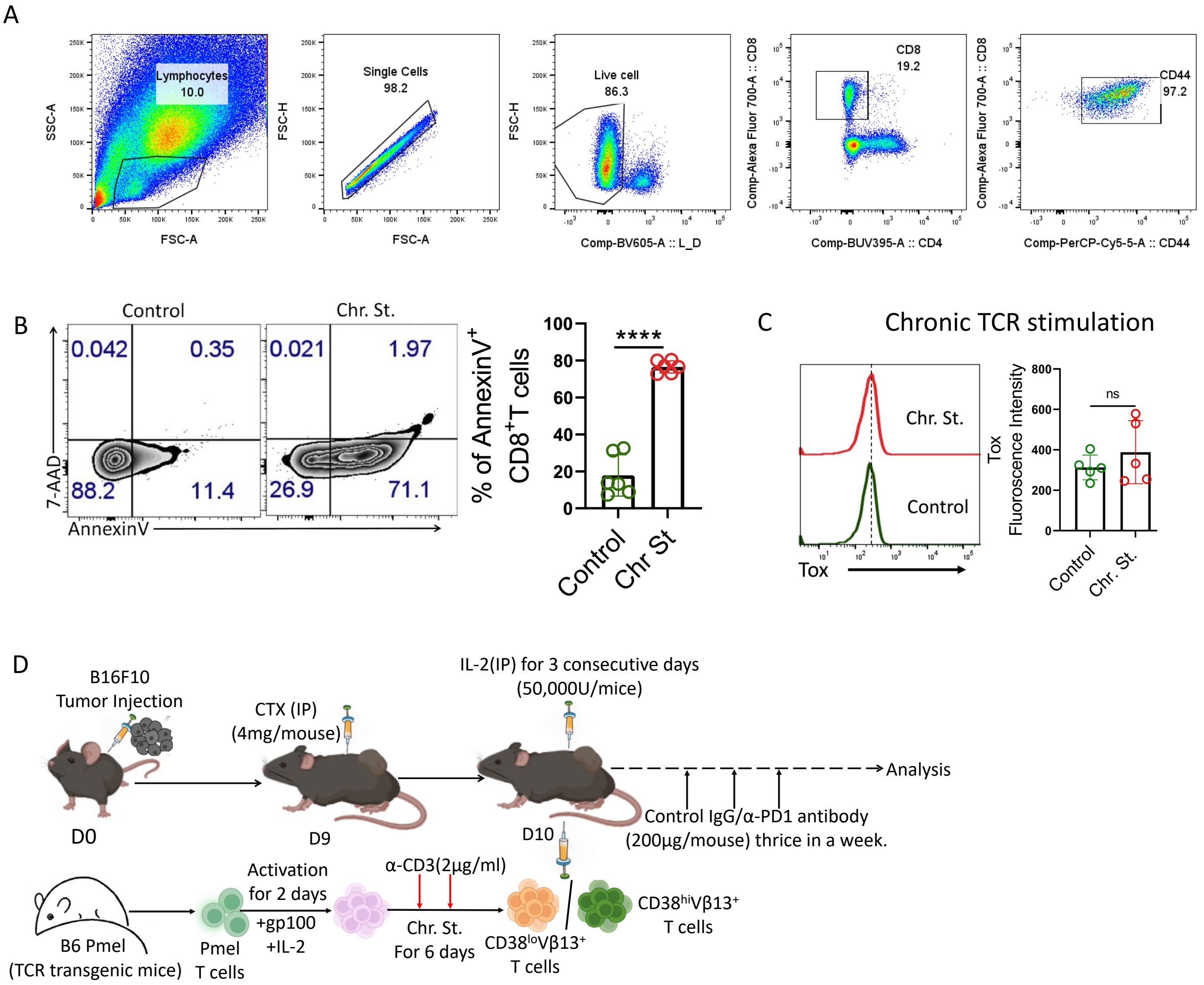

A
Lymphocytes 10.0
SSC-A
FSC-A
Single Cells 98.2
FSC-H
FSC-A
Live cell 86.3
FSC-H
Comp-BV605-A :: L_D
CD8 19.2
Comp-Alexa Fluor 700-A :: CD8
Comp-BUV395-A :: CD4
CD44 97.2
Comp-Alexa Fluor 700-A :: CD8
Comp-PerCP-Cy5-5-A :: CD44
B
Control
Chr. St.
0.042
0.35
88.2
11.4
0.021
1.97
26.9
71.1
7-AAD
AnnexinV
% of AnnexinV+ CD8+T cells
****
Control
Chr St
C
Chronic TCR stimulation
Chr. St.
Control
Tox
Tox Fluoroscence Intensity
ns
Control
Chr. St.
D
B16F10 Tumor Injection
CTX (IP) (4mg/mouse)
IL-2(IP) for 3 consecutive days (50,000U/mice)
Analysis
Control IgG/α-PD1 antibody (200μg/mouse) thrice in a week.
D0
D9
D10
B6 Pmel (TCR transgenic mice)
Pmel T cells
Activation for 2 days
+gp100 +IL-2
α-CD3(2μg/ml)
Chr. St. For 6 days
CD38loVβ13+ T cells
CD38hiVβ13+ T cells

Fig. S1. Characterization of $CD8^+$ T cells from tumor site and chronically stimulated *in vitro*. (A) Gating strategy used for flow cytometry analysis of tumor samples. (B) Flow cytometric analysis of apoptotic cell death of control and chronically stimulated $CD8^+$ T cells. The adjacent bar represents cumulative data from six independent experiments. (C) Expression of Tox in *in vitro* chronically stimulated $CD8^+$ T cell. The adjacent bar represents cumulative data of mean fluorescence intensity from five independent experiments. (D) Schematic representation of the experimental strategies of the tumor control experiment of mice bearing B16-F10 melanoma tumor by transferring $CD38^{hi}$ and $CD38^{lo}$ Pmel T cells with either control IgG or anti-PD1 antibody. *, $P < 0.05$; **, $P < 0.01$; ***, $P < 0.005$; ****, $P < 0.0001$; ns, nonsignificant.

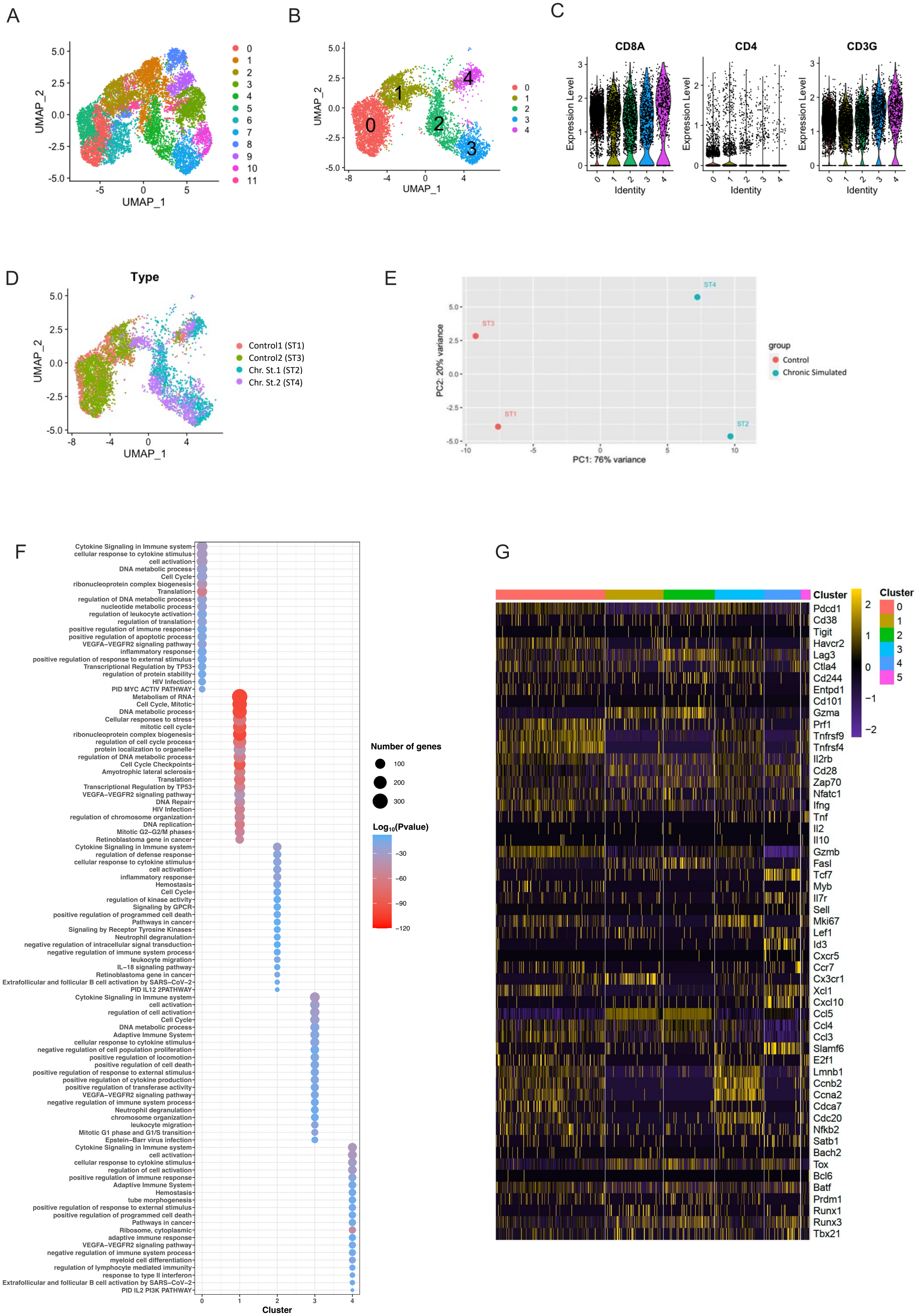
A
UMAP_1
UMAP_2
0
1
2
3
4
5
6
7
8
9
10
11
B
UMAP_1
UMAP_2
C
CD8A
CD4
CD3G
Expression Level
Identity
D
Type
UMAP_1
UMAP_2
Control1 (ST1)
Control2 (ST3)
Chr. St.1 (ST2)
Chr. St.2 (ST4)
E
ST1
ST2
ST3
ST4
PC1: 76% variance
PC2: 20% variance
group
Control
Chronic Simulated
F
Cytokine Signaling in Immune system
cellular response to cytokine stimulus
cell activation
DNA metabolic process
Cell Cycle
ribonucleoprotein complex biogenesis
Translation
regulation of DNA metabolic process
nucleotide metabolic process
regulation of leukocyte activation
regulation of translation
positive regulation of immune response
positive regulation of apoptotic process
VEGFA−VEGFR2 signaling pathway
inflammatory response
positive regulation of response to external stimulus
Transcriptional Regulation by TP53
regulation of protein stability
HIV Infection
PID MYC ACTIV PATHWAY
Metabolism of RNA
Cell Cycle, Mitotic
DNA metabolic process
Cellular responses to stress
mitotic cell cycle
ribonucleoprotein complex biogenesis
regulation of cell cycle process
protein localization to organelle
regulation of DNA metabolic process
Cell Cycle Checkpoints
Amyotrophic lateral sclerosis
Translation
Transcriptional Regulation by TP53
VEGFA−VEGFR2 signaling pathway
DNA Repair
HIV Infection
regulation of chromosome organization
DNA replication
Mitotic G2−G2/M phases
Retinoblastoma gene in cancer
Cytokine Signaling in Immune system
regulation of defense response
cellular response to cytokine stimulus
cell activation
inflammatory response
Hemostasis
Cell Cycle
regulation of kinase activity
Signaling by GPCR
positive regulation of programmed cell death
Pathways in cancer
Signaling by Receptor Tyrosine Kinases
Neutrophil degranulation
negative regulation of intracellular signal transduction
negative regulation of immune system process
leukocyte migration
IL−18 signaling pathway
Retinoblastoma gene in cancer
Extrafollicular and follicular B cell activation by SARS−CoV−2
PID IL12 2PATHWAY
Cytokine Signaling in Immune system
cell activation
regulation of cell activation
Cell Cycle
DNA metabolic process
Adaptive Immune System
cellular response to cytokine stimulus
negative regulation of cell population proliferation
positive regulation of locomotion
positive regulation of cell death
positive regulation of response to external stimulus
positive regulation of cytokine production
positive regulation of transferase activity
VEGFA−VEGFR2 signaling pathway
negative regulation of immune system process
Neutrophil degranulation
chromosome organization
leukocyte migration
Mitotic G1 phase and G1/S transition
Epstein−Barr virus infection
Cytokine Signaling in Immune system
cell activation
cellular response to cytokine stimulus
regulation of cell activation
positive regulation of immune response
Adaptive Immune System
Hemostasis
tube morphogenesis
positive regulation of response to external stimulus
positive regulation of programmed cell death
Pathways in cancer
Ribosome, cytoplasmic
adaptive immune response
VEGFA−VEGFR2 signaling pathway
negative regulation of immune system process
myeloid cell differentiation
regulation of lymphocyte mediated immunity
response to type II interferon
Extrafollicular and follicular B cell activation by SARS−CoV−2
PID IL2 PI3K PATHWAY
Cluster
Number of genes
100
200
300
Log10(Pvalue)
−30
−60
−90
−120
G
Cluster
Pdcd1
Cd38
Tigit
Havcr2
Lag3
Ctla4
Cd244
Entpd1
Cd101
Gzma
Prf1
Tnfrsf9
Tnfrsf4
Il2rb
Cd28
Zap70
Nfatc1
Ifng
Tnf
Il2
Il10
Gzmb
Fasl
Tcf7
Myb
Il7r
Sell
Mki67
Lef1
Id3
Cxcr5
Ccr7
Cx3cr1
Xcl1
Cxcl10
Ccl5
Ccl4
Ccl3
Slamf6
E2f1
Lmnb1
Ccnb2
Ccna2
Cdca7
Cdc20
Nfkb2
Satb1
Bach2
Tox
Bcl6
Batf
Prdm1
Runx1
Runx3
Tbx21

Fig. S2: Single-cell RNA sequencing analysis of exhausted CD8$^+$ T cells. (A) Uniform manifold approximation and projection (UMAP) visualization of the scRNA-seq clusters of T cells (n = 12,071) from 4 samples (ST1, ST2, ST3, ST4). (B) UMAP showing scRNA-seq clusters of CD8 (n=6,710) from aforementioned samples. (C) Violin plot shows expression of CD8A (Marker for CD8$^+$ T cell), CD4, CD3G (Pan T cell marker) genes across the scRNA-seq clusters of CD8$^+$ T cells. (D) UMAP visualization showing the origin of the scRNA-seq clusters of CD8$^+$ T cells. (E) Principal-component analysis (PCA) plot of aggregated signal in scRNA-seq data. Single-cell level measurements for each sample were aggregated to obtain the PCA analysis, related to the UMAP plot in (A). (F) Gene ontology analysis. The bubble graph displays the 20 most significantly enriched pathways by log(p-value) for each chronic stimulated CD8$^+$ T cell cluster established in Figure 3A. (G) Heatmap showing log2 fold change expression of selected genes across CD8$^+$ T cell clusters established in figure 3E.

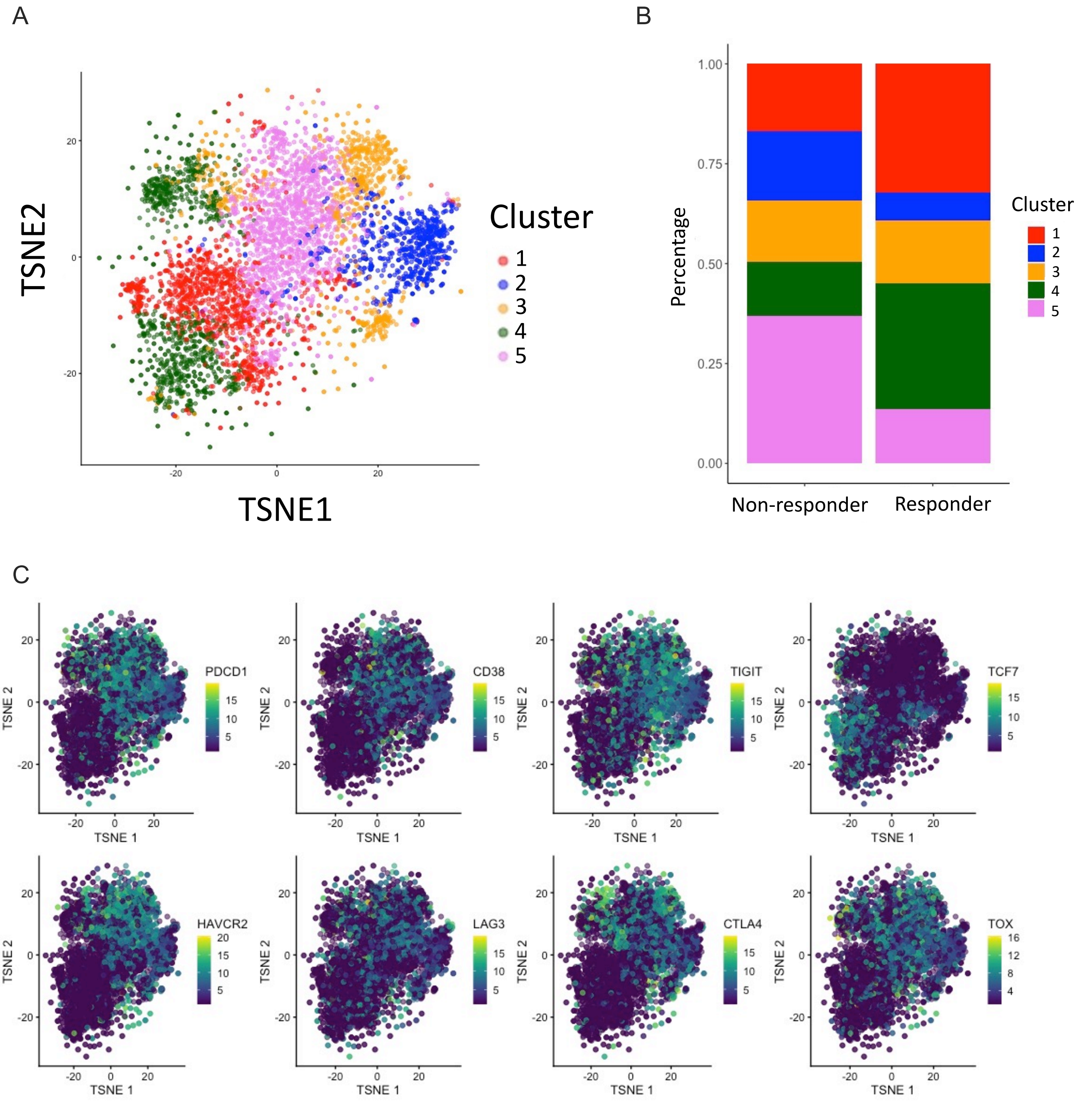
A
TSNE2
TSNE1
Cluster
1
2
3
4
5
B
Percentage
Non-responder
Responder
Cluster
1
2
3
4
5
C
PDCD1
CD38
TIGIT
TCF7
HAVCR2
LAG3
CTLA4
TOX
TSNE 1
TSNE 2

Fig. S3: Single-cell RNA sequencing analysis of CD8$^+$ T cells from metastatic melanoma patients. (A) tSNE plot showing clusters of CD8$^+$ T cells obtained from 32 patients (n=48) bearing metastatic melanoma (B) Bar plot showing the percentage distribution of CD8$^+$ T cells among clusters between non-responders and responders. (C) Single-cell gene expression levels of representative genes illustrated in the tSNE plot. Gene expressions are color-coded: Yellow-Green, expressed; black, not expressed.

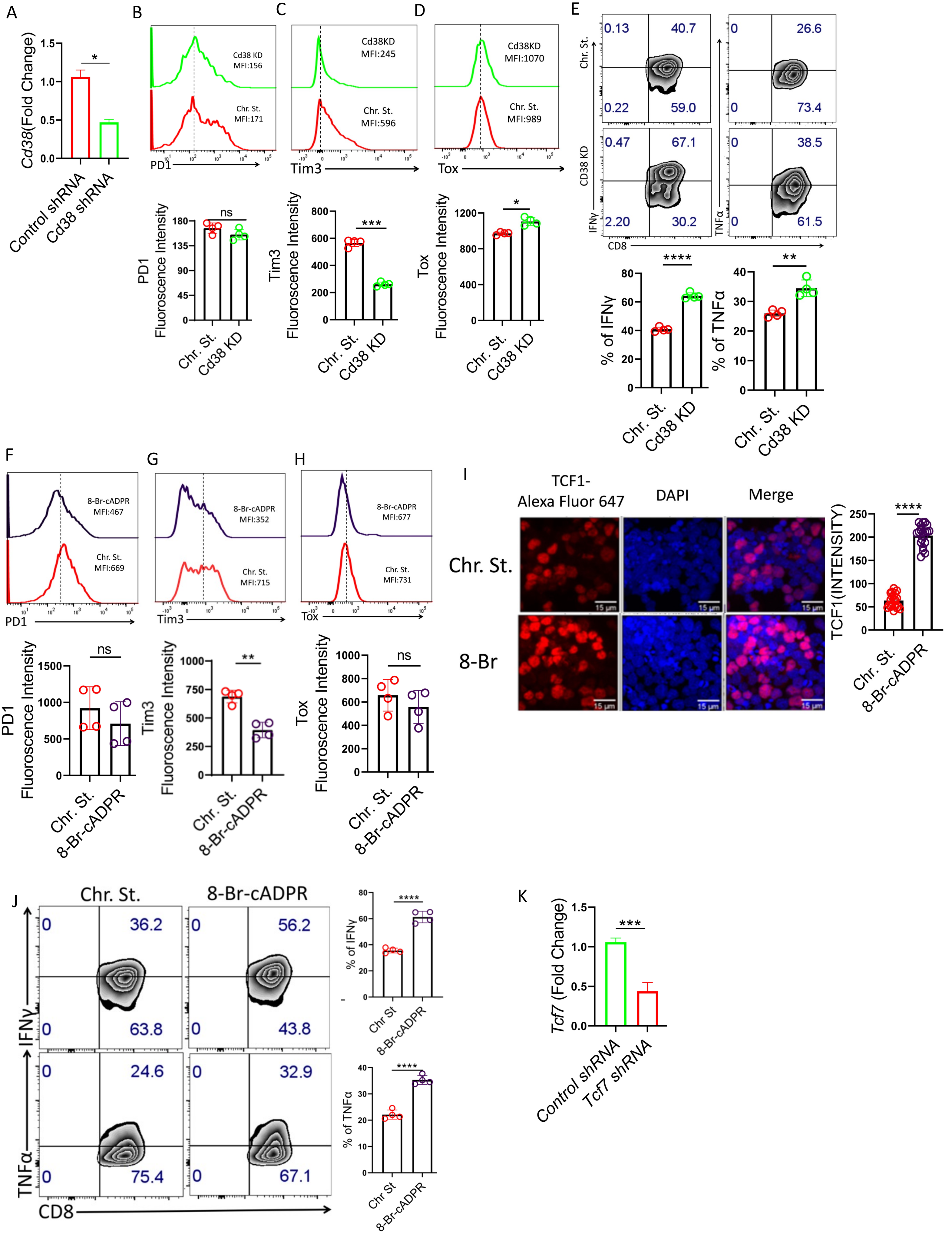
A
Cd38(Fold Change)
Control shRNA
Cd38 shRNA
*
B
Cd38 KD MFI:156
Chr. St. MFI:171
PD1
C
Cd38KD MFI:245
Chr. St. MFI:596
Tim3
D
Cd38KD MFI:1070
Chr. St. MFI:989
Tox
PD1 Fluoroscence Intensity
ns
Tim3 Fluoroscence Intensity
***
Tox Fluoroscence Intensity
*
Chr. St.
Cd38 KD
E
Chr. St.
0.13
40.7
0.22
59.0
0
26.6
0
73.4
CD38 KD
0.47
67.1
2.20
30.2
0
38.5
0
61.5
IFNγ
TNFα
CD8
% of IFNγ
****
% of TNFα
**
F
8-Br-cADPR MFI:467
Chr. St. MFI:669
PD1
G
8-Br-cADPR MFI:352
Chr. St. MFI:715
Tim3
H
8-Br-cADPR MFI:677
Chr. St. MFI:731
Tox
PD1 Fluoroscence Intensity
ns
Tim3 Fluoroscence Intensity
**
Tox Fluoroscence Intensity
ns
Chr. St.
8-Br-cADPR
I
TCF1-Alexa Fluor 647
DAPI
Merge
Chr. St.
8-Br
15 μm
TCF1(INTENSITY)
****
Chr. St.
8-Br-cADPR
J
Chr. St.
8-Br-cADPR
0
36.2
0
63.8
0
56.2
0
43.8
0
24.6
0
75.4
0
32.9
0
67.1
IFNγ
TNFα
CD8
% of IFNγ
****
% of TNFα
****
Chr St
8-Br-cADPR
K
Tcf7 (Fold Change)
***
Control shRNA
Tcf7 shRNA

Fig. S4. Inhibition of CD38 improves the functionality of chronically exhausted CD8$^+$ T cells. (A) Transcript levels of *Cd38* in chronically stimulated T cells transduced with either control shRNA or Cd38 shRNA. The bar represents cumulative data from four independent experiments. (B-E) Expression of (B) PD1, (C) Tim3, (D) Tox, and (E) production of intracellular cytokines (IFNγ and TNFα) in chronically expanded CD8$^+$ T cells transduced with either control shRNA or Cd38 shRNA. The bar diagrams (bottom panels) represent cumulative data of (B) median fluorescence intensity, (C-E) mean fluorescence intensity, and (E) frequency of cytokine positive cells from four independent experiments. (F-H) Expression of (F) PD1, (G) Tim3, and (H) Tox in chronically expanded CD8$^+$ T cells treated with either vehicle control or 8-Br-cADPR. The bar diagrams (bottom panels) represent cumulative data of mean fluorescence intensity (F & H), and (G) median fluorescence intensity from four independent experiments. (I) Confocal microscopic image of chronically stimulated T cells treated with either vehicle control or 8-Br-cADPR and stained with TCF1 (in red) nucleus stained with DAPI (in blue). (J) Intracellular cytokines (IFNγ and TNFα) in chronically expanded CD8$^+$ T cells treated with either vehicle control or 8-Br-cADPR. Adjacent bars represent cumulative data from four independent experiments. (K) Transcript levels of *Tcf7* in chronically stimulated T cells transduced with either control shRNA or Tcf7 shRNA. The bar represents cumulative data from three independent experiments. *, $P < 0.05$; **, $P < 0.01$; ***, $P < 0.005$; ****, $P < 0.0001$; ns, nonsignificant.

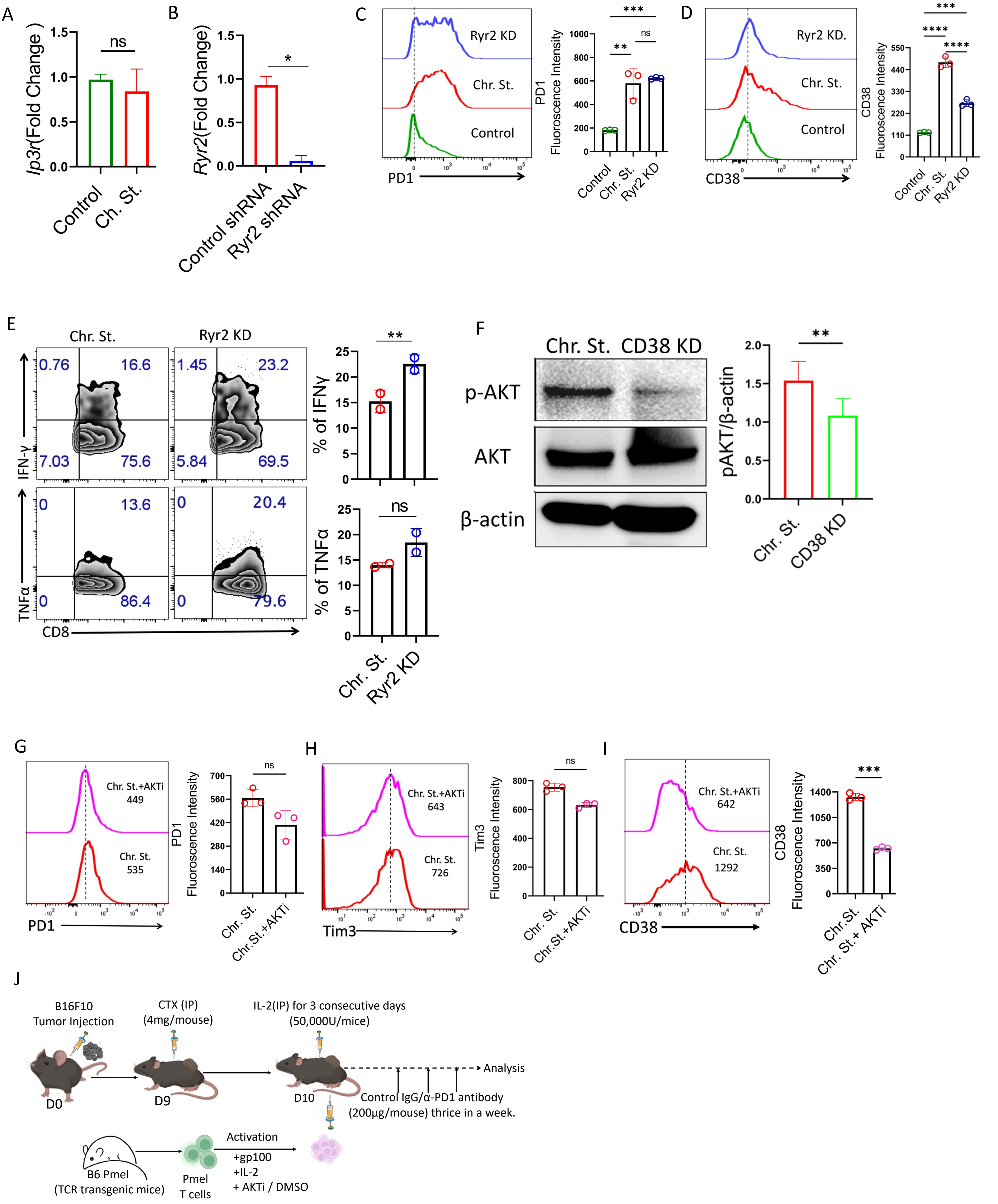
A
Ip3r(Fold Change)
ns
Control
Ch. St.
B
Ryr2(Fold Change)
*
Control shRNA
Ryr2 shRNA
C
Ryr2 KD
Chr. St.
Control
PD1
PD1 Fluorescence Intensity
***
**
ns
Control
Chr. St.
Ryr2 KD
D
Ryr2 KD.
Chr. St.
Control
CD38
CD38 Fluorescence Intensity
***
****
****
Control
Chr. St.
Ryr2 KD
E
Chr. St.
Ryr2 KD
0.76
16.6
7.03
75.6
1.45
23.2
5.84
69.5
0
13.6
0
86.4
0
20.4
0
79.6
IFN-γ
TNFα
CD8
% of IFNγ
**
% of TNFα
ns
Chr. St.
Ryr2 KD
F
Chr. St.
CD38 KD
p-AKT
AKT
β-actin
pAKT/β-actin
**
Chr. St.
CD38 KD
G
Chr. St.+AKTi
449
Chr. St.
535
PD1
PD1 Fluorescence Intensity
ns
Chr. St.
Chr.St.+AKTi
H
Chr. St.+AKTi
643
Chr. St.
726
Tim3
Tim3 Fluorescence Intensity
ns
Chr. St.
Chr.St.+AKTi
I
Chr. St.+AKTi
642
Chr. St.
1292
CD38
CD38 Fluorescence Intensity
***
Chr.St.
Chr. St.+ AKTi
J
B16F10
Tumor Injection
CTX (IP)
(4mg/mouse)
IL-2(IP) for 3 consecutive days
(50,000U/mice)
Analysis
D0
D9
D10
Control IgG/α-PD1 antibody
(200μg/mouse) thrice in a week.
B6 Pmel
(TCR transgenic mice)
Pmel
T cells
Activation
+gp100
+IL-2
+ AKTi / DMSO

Fig. S5. **Functional and phenotypic assessment of chronically exhausted CD8⁺ T cells upon RyR2 or AKT inhibition.** (A) Transcript levels of *Ip3r* in control and chronically stimulated T cells. Data is representative of four independent experiments. (B) Transcript levels of *Ryr2* in chronically stimulated T cells transduced with either control shRNA or Ryr2 shRNA. The bar represents cumulative data from three independent experiments. (C-D) Expression of (C) PD1, and (D) CD38 in control and chronically expanded $CD8^+$ T cells transduced with either control shRNA or Ryr2 shRNA. Adjacent bar diagrams represent cumulative data of (C) median fluorescence intensity and (D) mean fluorescence intensity from three independent experiments. (E) Intracellular cytokines (IFNγ and TNFα) in chronically expanded $CD8^+$ T cells transduced with either control shRNA or Ryr2 shRNA. Adjacent bar diagrams represent cumulative data from two independent experiments. (F) Expression of p-AKT level (Ser^{473}) in chronically stimulated $CD8^+$ T cells transduced with either control shRNA or CD38 shRNA evaluated by western blot. Adjacent bars represent data from three independent experiments. (G-I) Expression of (G) PD1, (H) Tim3, and (I) CD38 in chronically expanded $CD8^+$ T cells treated with or without AKTi. Adjacent bars represent cumulative data of mean fluorescence intensity from three independent experiments. (J) Schematic representation of the ACT protocol. *, $P < 0.05$; **, $P < 0.01$; ***, $P < 0.005$; ****, $P < 0.0001$; ns, nonsignificant.
